# A novel effective bio-originated methylene blue adsorbent: the porous biosilica from three marine diatom strains of *Nanofrustulum* spp. (Bacillariophyta)

**DOI:** 10.1038/s41598-023-36408-6

**Published:** 2023-06-06

**Authors:** Aleksandra Golubeva, Piya Roychoudhury, Przemysław Dąbek, Jagoda Pałczyńska, Oleksandra Pryshchepa, Piotr Piszczek, Paweł Pomastowski, Michał Gloc, Renata Dobrucka, Agnieszka Feliczak-Guzik, Izabela Nowak, Krzysztof J. Kurzydłowski, Bogusław Buszewski, Andrzej Witkowski

**Affiliations:** 1 Institute of Marine and Environmental Sciences, University of Szczecin, Mickiewicza 16a, 70-383 Szczecin, Poland; 2 Department of Inorganic and Coordination Chemistry, Faculty of Chemistry, Nicolaus Copernicus University, Gagarina 7, 87-100 Toruń, Poland; 3 Centre for Modern Interdisciplinary Technologies, Nicolaus Copernicus University, Wileńska 4, 87-100 Toruń, Poland; 4 Faculty of Materials Science and Engineering, Warsaw University of Technology, Wołoska 141, 02-507 Warsaw, Poland; 5 Department of Industrial Products and Packaging Quality, Institute of Quality Science, Poznań University of Economics and Business, al. Niepodległości 10, 61-875 Poznan, Poland; 6 Department of Applied Chemistry, Faculty of Chemistry, Adam Mickiewicz University, Uniwersytetu Poznańskiego 8, 61-614 Poznań, Poland; 7 Faculty of Mechanical Engineering, Bialystok University of Technology, ul. Wiejska 45 c, 15-351 Bialystok, Poland; 8 Department of Environmental Chemistry and Bioanalysis, Faculty of Chemistry, Nicolaus Copernicus University, Gagarina 7, 87-100 Toruń, Poland; 9 Prof. Jan Czochralski Kuyavian-Pomeranian Research and Development Centre, Krasińskiego 4, 87-100 Toruń, Poland

**Keywords:** Pollution remediation, Bioinspired materials

## Abstract

In the present paper, for the first time the ability of the porous biosilica originated from three marine diatom strains of ‘*Nanofrustulum spp.’* viz. *N. wachnickianum* (SZCZCH193), *N. shiloi* (SZCZM1342), *N.* cf. *shiloi* (SZCZP1809), to eliminate MB from aqueous solutions was investigated. The highest biomass was achieved under silicate enrichment for *N. wachnickianum* and *N. shiloi* (0.98 g L^−1^ DW and 0.93 g L^−1^ DW respectively), and under 15 °C for *N*. cf. *shiloi* (2.2 g L^−1^ DW). The siliceous skeletons of the strains were purified with hydrogen peroxide and characterized by SEM, EDS, the N_2_ adsorption/desorption, XRD, TGA, and ATR-FTIR. The porous biosilica (20 mg DW) obtained from the strains i.e. SZCZCH193, SZCZM1342, SZCZP1809, showed efficiency in 77.6%, 96.8%, and 98.1% of 14 mg L^−1^ MB removal under pH 7 for 180 min, and the maximum adsorption capacity was calculated as 8.39, 19.02, and 15.17 mg g^−1^, respectively. Additionally, it was possible to increase the MB removal efficiency in alkaline (pH = 11) conditions up to 99.08% for SZCZP1809 after 120 min. Modelling revealed that the adsorption of MB follows Pseudo-first order, Bangham’s pore diffusion and Sips isotherm models.

## Introduction

Diatoms (Bacillariophyta), representing a major group of photosynthetic microorganisms, are unicellular eukaryotic microalgae that live within cell walls composed of 3D structured porous biosilica (SiO_2_). They play an essential role in global carbon and silicon cycles in the ocean and their photosynthetic activity accounts for almost one-fifth of the Earth’s primary productivity^1,2^. Diatoms attract increasing attention in the applied sciences due to their potential for producing a variety of bioactive compounds and fine chemicals for industrial applications: fucoxanthin is known for its antioxidant effect and can be used in pharmaceuticals and cosmetics^3^; unsaturated fatty acids have been used as food supplements^4^; triacylglycerols (TAG) provide a carbon feedstock for conversion to biofuels^5^. The natural porous architecture of diatomaceous frustules gained the attention in a field of drug delivery^6^, biosensing^7^, and metal recovery^8^. Diatoms have enormous biotechnological potential for biorefinery processes^9^, thereby their biomass could be used in the production of various compounds in a cost-effective way.

The widespread use of various organic pollutants, e.g. drugs^10^, antibiotics^11^, phenols^12^, and dyes^13^, in industry has resulted in the problem of water pollution. They are stored as industrial wastes and then purged into environmental water bodies, changing colorless clean water into contaminated colored wastes. Water-soluble basic dyes are commonly used in paper, polyester, silk, cotton, and wool coloration^14^. This contamination is highly toxic and could negatively affect humans, causing breathing problems, eye damage, and methemoglobinemia^15–17^. Methylene blue (MB) is known as a model dye that is used to evaluate the removal capacity of different materials and an indicator of the mesoporous nature of adsorbents^18^.

Currently, numerous studies have been done to find an efficient green dye removal method, so that the dye in wastewater could be recovered. One of the most promising degradation methods is adsorption, which gives better results, could be used for different types of dyes, does not require highly sophisticated equipment, insensitive to toxic co-pollutants in wastewaters, and does not produce toxic substances^19^. Activated carbon, the most commonly applied natural adsorbent, has been used in numerous studies and showed high adsorption capacity in the removal of MB, although the high cost and difficult regeneration process resulted in a further search to find low-cost and highly effective adsorbents^20^. Many non-conventional adsorbents, especially those based on natural products, have been proposed as adsorption agents. High adsorption capacities have been shown for bioadsorbents (dead and live biomass of bacteria^21^, algae^22^, fungi^23^, plants^24^, and agricultural wastes^25^), zeolites^26^ and diatomite^27^. To the best of our knowledge, only a few studies have been performed with pure diatomaceous biosilica extracted from *Punnularia* sp.^28^ and *Cyclotella* sp.^29^, with a greater focus on metal-doped diatomaceous silica^30,31^, diatomaceous earth^27,32^ and chemically synthesized mesoporous silica^33,34^. Although chemically synthesized silica demonstrates high adsorption efficiency, some investigations suggested that this material can show cytotoxicity^35,36^, while diatom-originated biosilica reported to be non-cytotoxic material^37^, therefore could be used in non-damaging way. In the present study, porous biosilica from three different marine diatom strains of the genus *Nanofrustulum* Round, Hallsteinsen & Paasche grown in the Szczecin Diatom Culture Collection (SZCZ), University of Szczecin, Institute of Marine and Environmental Sciences, Poland, has been for the first time characterized and identified as highly effective and cheap MB adsorbent.

## Results

### Batch cultivation of diatom cultures

**Figure 1:**
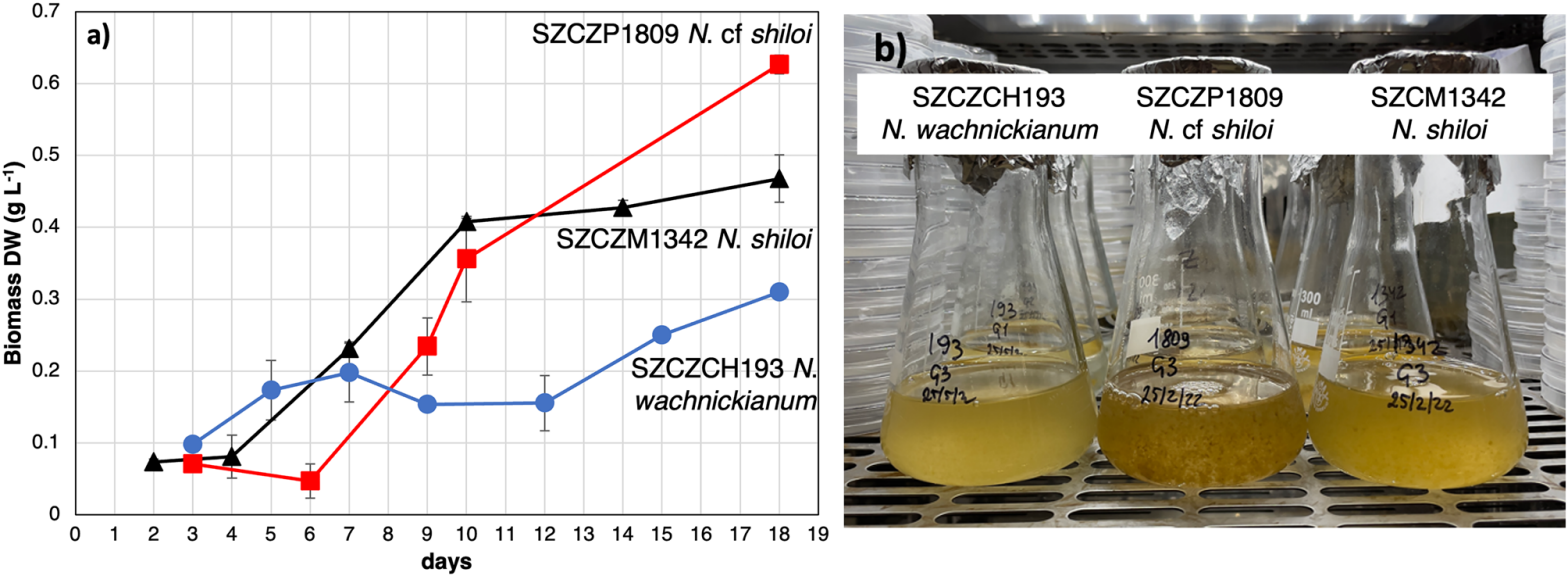
Batch culture of SZCZCH193 *N*. *wachnickianum*, SZCZM1342 *N. shiloi*, SZCZP1809 *N.* cf. *shiloi*: (**a**) growth dynamics of biomass DW; (**b**) cultivation flasks.

Three strains of *Nanofrustulum* spp. showed distinct lag, exponential, and stationary phases during batch culture (Fig. 1a,b). The lag phase of the strain SZCZCh193 *N. wachnickianum* was observed until the 5th day of cultivation, with exponential growth for 7 days from the 5th to 12th day, and a stationary phase starting from the 12th day of growth. The maximum specific growth rate of the exponential phase was calculated as 0.115 d^−1^ (R^2^ = 0.95). The growth of strain SZCZM1342 *N. shiloi* showed a lag (until the 4th day), exponential (for 6 days, from 4 to 10th day), and stationary (from 10th day) phases, and the maximum specific growth rate of the exponential phase was calculated as 0.270 d^−1^ (R^2^ = 0.97) 6 days after inoculation, SZCZP1809 *N.* cf. *shiloi* showed exponential growth for 10 days (to the 16th day of cultivation), with a following stationary phase of growth. The maximum specific growth rate during the exponential phase was calculated as 0.513 d^−1^ (R^2^ = 0.99). Therefore, it can be said that the maximum biomass yields for SZCZCh193 *N. wachnickianum* (0.39 ± 0.039 g L^−1^ DW), and SZCZM1342 *N. shiloi* (0.47 ± 0.033 g L^−1^ DW) were obtained on the 18th day, and for SZCZP1809 *N.* cf. *shiloi* (0.63 ± 0.013 g L^−1^ DW) on the 16th day of cultivation.

Experiments with different salinities of artificial seawater Guillard’s f/2 medium (Table 1, Supplementary Figure S1a) showed that low salinities (15 and 20‰) resulted in lower biomass accumulation than high salinities (35 and 45‰). The highest dry biomass yield was observed with 45‰ for SZCZCH193* N*. *wachnickianum* (n = 8, one-way ANOVA *p* = 0.0319; Turkey HSD *p* = 0.0251 between 35 and 45‰) and strains SZCZP1809 *N.* cf. *shiloi*, (n = 8, one-way ANOVA *p* = 0.002; Turkey HSD *p* = 0.0361 between 35 and 45%) and no significant increase in biomass yield for SZCZM1342 *N. shiloi* (n = 8, one-way ANOVA *p* = 0.0003; Turkey HSD *p* = 0.6117 between 35 and 45%).

**Table 1:** Dry biomass accumulation (g L^−1^) under a variety of cultivation parameters (temperature, light intensity, salinity, and nitrate, phosphate, silicate concentrations in Guillard′s f/2 medium) in the late exponential phase of batch cultivation of three *Nanofrustulum* spp strains (mean ± standard deviation).

Cultivation parameter	Nitrate, mM L^−1^					Phosphate, mM L^−1^				
	0.88	4.41	8.82	13.23	17.64	0.04	0.18	0.36	0.54	0.72
SZCZCH193	0.499 ± 0.064	0.473 ± 0.023	0.463 ± 0.037	0.452 ± 0.021	0.430 ± 0.004	0.531 ± 0.137	0.633 ± 0.077	0.445 ± 0.004	0.491 ± 0.057	0.463 ± 0.017
SZCZM1342	0.502 ± 0.091	0.591 ± 0.080	0.524 ± 0.019	0.623 ± 0.029	0.647 ± 0.007	0.622 ± 0.007	0.701 ± 0.025	0.710 ± 0.056	0.742 ± 0.014	0.686 ± 0.048
SZCZP1809	0.774 ± 0.058	1.01 ± 0.095	0.759 ± 0.169	0.609 ± 0.016	0.683 ± 0.006	0.735 ± 0.026	0.699 ± 0.013	0.791 ± 0.093	0.732 ± 0.063	0.735 ± 0.000

Cultivation parameter	Silicate, mM L^−1^					Temperature, °C				
	0.11	0.53	1.06	1.59	2.12	15	20	30		
SZCZCH193	0.447 ± 0.089	0.466 ± 0.110	0.711 ± 0.004	0.807 ± 0.030	0.977 ± 0.004	0.331 ± 0.014	0.352 ± 0.002	0.865 ± 0.187		
SZCZM1342	0.402 ± 0.003	0.570 ± 0.057	0.636 ± 0.000	0.816 ± 0.040	0.930 ± 0.014	0.530 ± 0.034	0.568 ± 0.042	0.624 ± 0.011		
SZCZP1809	0.469 ± 0.090	0.600 ± 0.006	0.670 ± 0.059	0.810 ± 0.037	0.989 ± 0.047	2.208 ± 0.213	1.178 ± 0.074	0.758 ± 0.025		

Cultivation parameter	Light intensity, µmol s^−1^ m^−2^				Salinity, ‰					
	10	50	100	150	15	20	35	45		
SZCZCH193	0.308 ± 0.009	0.329 ± 0.050	0.383 ± 0.141	0.413 ± 0.093	0.306 ± 0.042	0.232 ± 0.041	0.352 ± 0.003	0.442 ± 0.059		
SZCZM1342	0.395 ± 0.116	0.718 ± 0.195	0.733 ± 0.088	0.718 ± 0.019	0.053 ± 0.007	0.116 ± 0.051	0.421 ± 0.001	0.387 ± 0.002		
SZCZP1809	0.557 ± 0.014	1.037 ± 0.043	0.900 ± 0.038	1.458 ± 0.286	0.282 ± 0.017	0.374 ± 0.039	0.566 ± 0.008	0.798 ± 0.093		

The following set of experiments (Table 1, Supplementary Figure S1b) showed a tendency of *Nanofrustulum* spp. to increase their biomass yield with increasing silicate concentration in Guillard’s f/2 medium 20 times (2.12 mM) for all three strains (SZCZCH193: n = 10, one-way ANOVA *p* = 0.0018, Turkey HSD *p* = 0.0024, 0.0028, 0.608, 0.2001 (between 2.12 and 0.11, 0.53, 1.06, 1.59 respectively); SZCZM1342: n = 10, one-way ANOVA *p* = 0.0008, Turkey HSD *p* = 0.001 (between 2.12 mM and 0.11, 0.53 or 1.06 mM) and *p* = 0.0731 (between 2.12 and 1.59 mM); SZCZP1809: n = 10, one-way ANOVA *p* = 0.0015, Turkey HSD *p* = 0.0012, 0.0046, 0.0111, 0.1035 (between 2.12 and 0.11, 0.53, 1.06, 1.59 respectively)).

Experiments with different illumination intensities (Table 1, Supplementary Figure S1e) showed that the high light intensity (150 µmol s^−1^ m^−2^) did not result in different biomass yields for SZCZCH193 *N*. *wachnickianum* and SZCZM1342 *N. shiloi*, and increased biomass accumulation for SZCZP1809 *N.* cf. *shiloi*. (SZCZCH193: n-8, one-way ANOVA *p* = 0.6473; SZCZM1342: n = 8, one-way ANOVA *p* = 0.1237; SZCZP1809: n = 8, one-way ANOVA *p* = 0.0155, Turkey HSD *p* = 0.0119 between 10 and 150 µmol s^−1^ m^−2^).

The impact of temperature (Table 1, Supplementary Figure S1f) on biomass accumulation was different for each strain. For SZCZCH193* N*. *wachnickianum*, the high temperature of cultivation (30 °C) increased the biomass yield (n = 6, one-way ANOVA *p* = 0.0259, Turkey HSD *p* = 0.032, 0.0357 between 20 and 15, 30 °C respectively); no differences in biomass accumulation for SZCZM1342 *N. shiloi* were observed (n = 6, one-way ANOVA *p* = 0.1296); and for SZCZP1809 *N.* cf. *shiloi* the low temperature of cultivation (15 °C) increased biomass yield (n = 6, one-way ANOVA *p* = 0.0027, Turkey HSD *p* = 0.0074, 0.0025 between 15 and 20, 30 °C respectively).

The variation in nitrate (Table 1, Supplementary Figure S1c) and phosphate (Table 1, Supplementary Figure S1d) concentrations in Guillard’s f/2 medium did not show differences in dry biomass yield for SZCZCH193 *N*. *wachnickianum* (nitrate: n = 10, one-way ANOVA *p* = 0.4732; phosphate: n = 10, one-way ANOVA *p* = 0.2371), SZCZM1342 *N. shiloi* (nitrate: n = 10, one-way ANOVA *p* = 0.1749; phosphate: n = 10, one-way ANOVA *p* = 0.1219), and SZCZP1809 *N.* cf. *shiloi* (nitrate: n = 10, one-way ANOVA *p* = 0.0455, Turkey HSD *p* = 0.0355 between 4.41 and 13.23 mM; phosphate: n = 10, one-way ANOVA *p* = 0.5765).

### Morphology and characterization of diatomaceous biosilica

**Figure 2:**
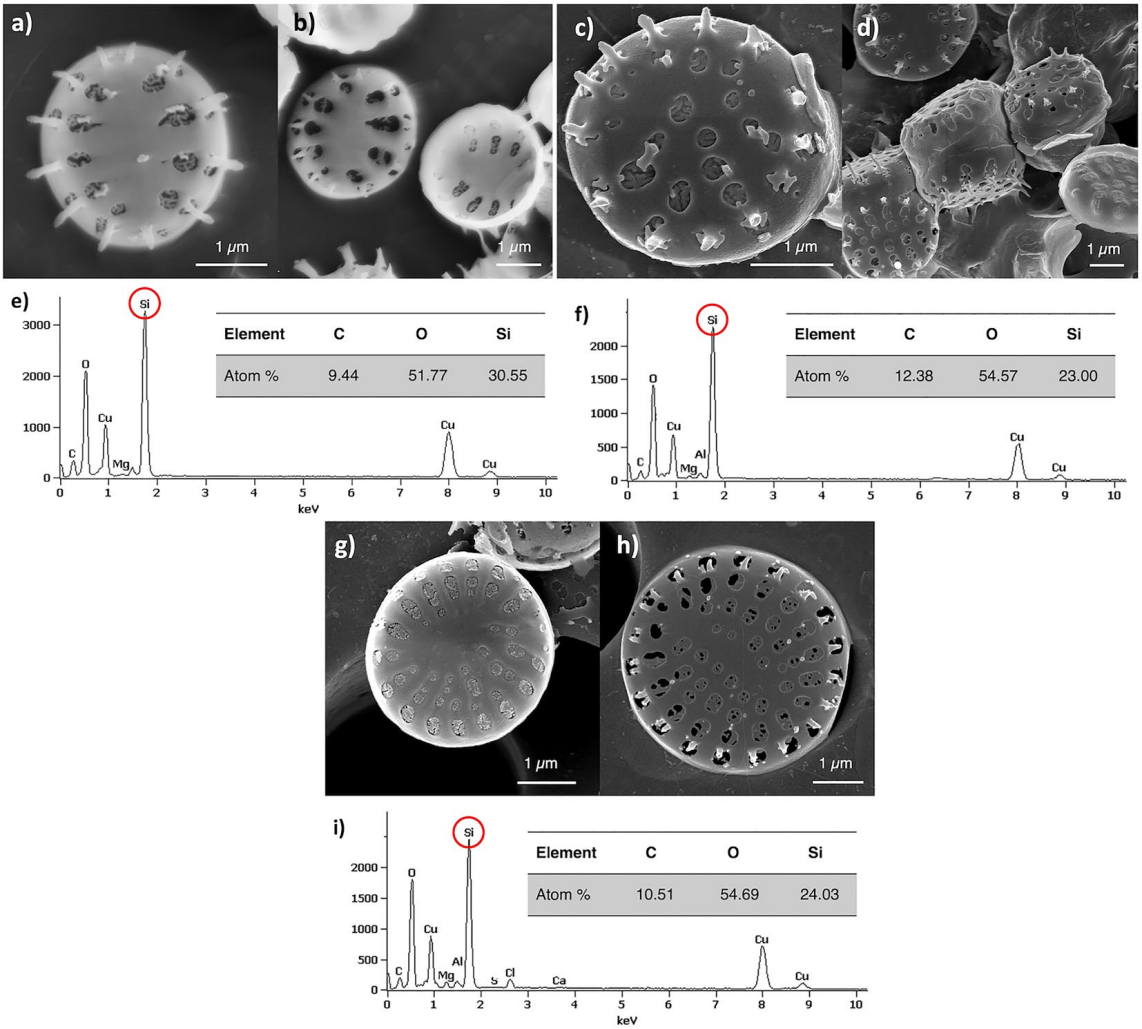
SEM images showing surface morphology of (**a**, **b**) SZCZCH193 *N*. *wachnickianum*, (**c**, **d**) SZCZM1342 *N. shiloi*, (**g**, **h**) SZCZP1809 *N.* cf. *shiloi,* and EDS spectra confirmed presence of Si and O on surface of (**e**) SZCZCH193 *N*. *wachnickianum*, (**f**) SZCZM1342 *N. shiloi*, (**i**) SZCZP1809 *N.* cf. *shiloi* (scale bar—1 µm, red circles show the presence of Si).

Scanning electron microscopy (SEM) revealed the similarities and differences in the morphology of the frustules of the three *Nanofrustulum* strains (Fig. 2a–d,g,h). The valves of all three strains’ frustules are oval. The length of the frustules slightly varies from 2.6–2.9 µm (SZCZCH193 *N. wachnickianum*) and 3.1–3.3 µm (SZCZM1342 *N. shiloi*) to 3.9–4.2 µm (SZCZP1809 *N.* cf. *shiloi*), and the width varies from 2.9–3.1 µm (SZCZCH193 *N. wachnickianum,* SZCZM1342 *N. shiloi*) to 3.6–4.0 µm (SZCZP1809 *N.* cf. *shiloi*). The sternum is broadly-lanceolate for SZCZCH193 *N. wachnickianum*, and narrow-linear for the other two strains. The pores on a valve face, called areolae, are slightly elongated towards the valve mantle, length and width were measured as 120–615 nm and 120–330 nm (SZCZCH193 *N. wachnickianum*); 150–450 nm and 120–400 nm (SZCZM1342 *N. shiloi*); 80–510 nm and 100–330 nm (SZCZP1809 *N.* cf. *shiloi*). There is only one row of areolae on the valve face of SZCZCH193 *N. wachnickianum* (Fig. 2a,b), then there are two rows on the valve face of SZCZM1342 *N. shiloi* (Fig. 2c,d) and up to four rows on the valve of SZCZP1809 *N.* cf*. shiloi* (Fig. 2g,h).

The energy-dispersive X-ray spectra (Fig. 2e,f,i) of the three strains showed distinct peaks for silicon (Si), oxygen (O), carbon (C), and copper (Cu), which is an effect of copper grids use in STEM imaging. The atomic percentage of major elements were recorded as 30.55, 24.03, and 23.00% of silicon, 51.77, 54.69, and 54.67% of oxygen, and 9.44, 10.51, and 12.38% of carbon for SZCZCH193 *N. wachickianum*, SZCZM1342 *N. shiloi*, SZCZP1809 *N.* cf. *shiloi* and respectively.

**Figure 3:**
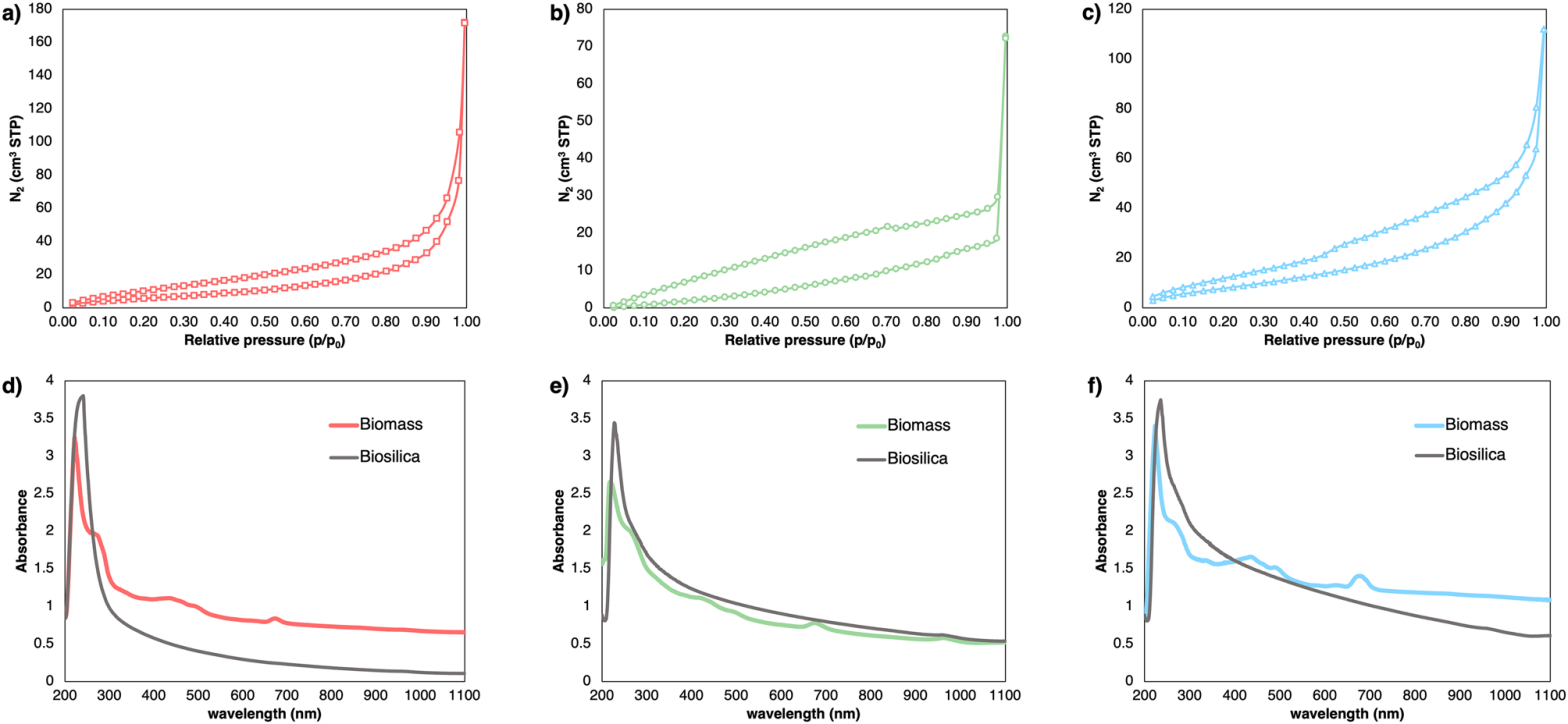
The N_2_ adsorption/desorption isotherms for (**a**) SZCZCH193* N*. *wachnickianum*, (**b**) SZCZM1342 *N. shiloi*, (**c**) SZCZP1809 *N.* cf. *shiloi*; and the UV–vis spectroscopy of biomass (colored line) and biosilica (gray line) for (**d**) SZCZCH193 *N*. *wachnickianum*, (**e**) SZCZM1342 *N. shiloi*, (**f**) SZCZP1809 *N.* cf. *shiloi*.

The specific surface area, pore volume, and pore diameter (Supplementary Table S1) of *Nanofrustulum* spp. strains frustules were estimated from the low-temperature N_2_ adsorption/desorption isotherms, presented in Fig. 3a–c, as 25.32 m^2^ g^−1^, 0.267 cm^3^ g^−1^, 4.22 nm respectively for SZCZCH193 *N. wachnickianium*, 21.78 m^2^ g^−1^, 0.113 cm^3^ g^−1^, 2.07 nm respectively for SZCZM1342 *N. shiloi*, and 35.23 m^2^ g^−1^, 0.174 cm^3^ g^−1^, 1.97 nm respectively for SZCZP1809 *N*. cf. *shiloi*.

The UV–vis spectroscopy of sonicated biomass showed a distinct peak at 230 nm and small peaks at 270, 430, 495 and 676 nm (Fig. 3d–f, green line). The pure, sonicated biosilica showed only one distinct peak at 230 nm (Fig. 3d–f, gray line).

**Figure 4:**
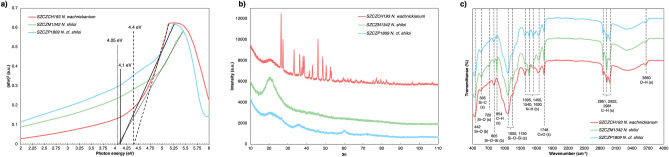
Characterization of pure diatomaceous biosilica with (**a**) Tauc’s plot—presented biosilica bandgaps; (**b**) XRD—showed amorphous structure of biosilica as well as the presence of quartz or inorganic salts; (**c**) FTIR spectra—revealed the presence of functional groups surrounding the surfaces of biosilica of SZCZCH193 *N*. *wachnickianum*, SZCZM1342 *N. shiloi*, SZCZP1809 *N.* cf. *shiloi* (s—stretching, b—bending; spectra are offset to aid comparison).

The biosilica Tauc’s plot (Fig. 4a) showed a steep linear increase in light absorption with increasing energy. The x-axis intersection point of the linear fit of the Tauc plot gives an estimate of the band gap energy of 4.40 eV for SZCZCH193 *N*. *wachnickianum*, 4.05 eV for SZCZM1342 *N. shiloi*, and 4.10 eV for SZCZP1809 *N.* cf. *shiloi.*

On the Fig. 4b XRD the results of X-ray diffraction analysis are presented. On the XRD pattern of all samples the broad peak at 2θ ≈ 15°–32°. However, for sample SZCZCH193 *N. wachnickianum* intense signals at 2θ ≈ 26.4°, 27.4°, 36.6°, 37.5°, 38.1°, 38.6°, 41.4°, 43.1°, 46.0°, 50.4°, 59.4°, 61.9°, 77.2° were distinguished, while the presence of low intensity signals at 2θ ≈ 26.4°, 36.4°, 46.2°, 48.7°, 60.7° were noticed for sample SZCZP1809 *N*. cf. *shiloi*.

ATR-FTIR spectra revealed distinct peaks (Fig. 4c) indicating the presence of O–H, C–H, C=O, N–H, and Si–O functional groups on the surface of frustules: 3,660 cm^−1^; 2981, 2922, and 2851 cm^−1^; 1748 cm^−1^; 1630, 1540, 1450, and 1395 cm^−1^; and 1150, 1050, 805, and 442 cm^−1^, respectively.

The PZC was calculated as the intercept between initial pH and change in pH value after 24 h of incubation and was estimated as pH_pzc_ = 6.0 for SZCZCH193* N*. *wachnickianum* and pH_pzc_ = 5.3 for SZCZM1342 *N. shiloi* and SZCZP1809 *N.* cf. *shiloi* (see Supplementary Figure S2a).

The low zeta potential values in the range of 2.0 – 4.0 pH for each sample can be seen on the graph (see Supplementary Figure S2b). In the pH range of 4.0 to 12.0 an increase of zeta potential is observed (zeta potential > –25 mV).

**Figure 5:**
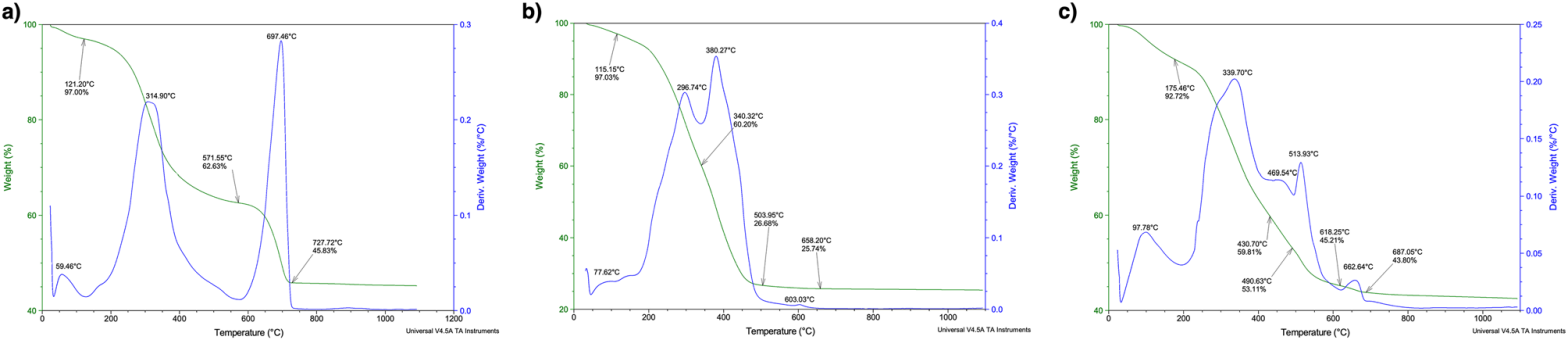
Thermal stability and transformation of pure diatomaceous biosilica (**a**) SZCZCH193 *N*. *wachnickianum*, (**b**) SZCZM1342 *N. shiloi*, (**c**) SZCZP1809 *N.* cf. *shiloi*.

Figure 5a–c presents the results of TGA analysis for all three biosilica samples. The plot of the mass loss against temperature did not allow us to derive more specific information about individual stage of the process. Thus, the differential thermal analysis (DTA) was performed which showed three main stages of the process (Fig. 5a–c). SZCZCH193 *N. wachnickianum* biosilica sample (Fig. 5a) showed three distinguished peaks on the DTA analysis plot appeared with maxima at 59.5, 314.9 and 697 °C. First stage ends up at 121.2 °C and mass loss of 3%, second one at 571.5 °C with mass loss of 34.6%, and third one at 727.72 °C with mass loss of 54.17%. Similarly, SZCZM1342 *N. shiloi* plot (Fig. 5b) revealed three stages in process with first one ending at 115 °C (mass loss of about 3%), second one at 340.32 °C with mass loss of 39.8%, and third one at 503 °C with mass loss of 73.32%. The DTA analysis revealed peaks at 77.62, 296.74, 380.27, and 603.03 °C. For sample SZCZP1809 *N.* cf. *shiloi* the most complex plot of DTA analysis has been observed (Fig. 5c) with peaks at 97.78, 339.70, 469.54, 513.93, and 662.64 °C. First stage ends at 175.46 °C with mass loss of 7.28%, second one at 490.6 °C with 46.89% mass loss, and third one at 687.05 °C with 56.92% mass loss.

### Methylene blue batch adsorption

**Figure 6:**
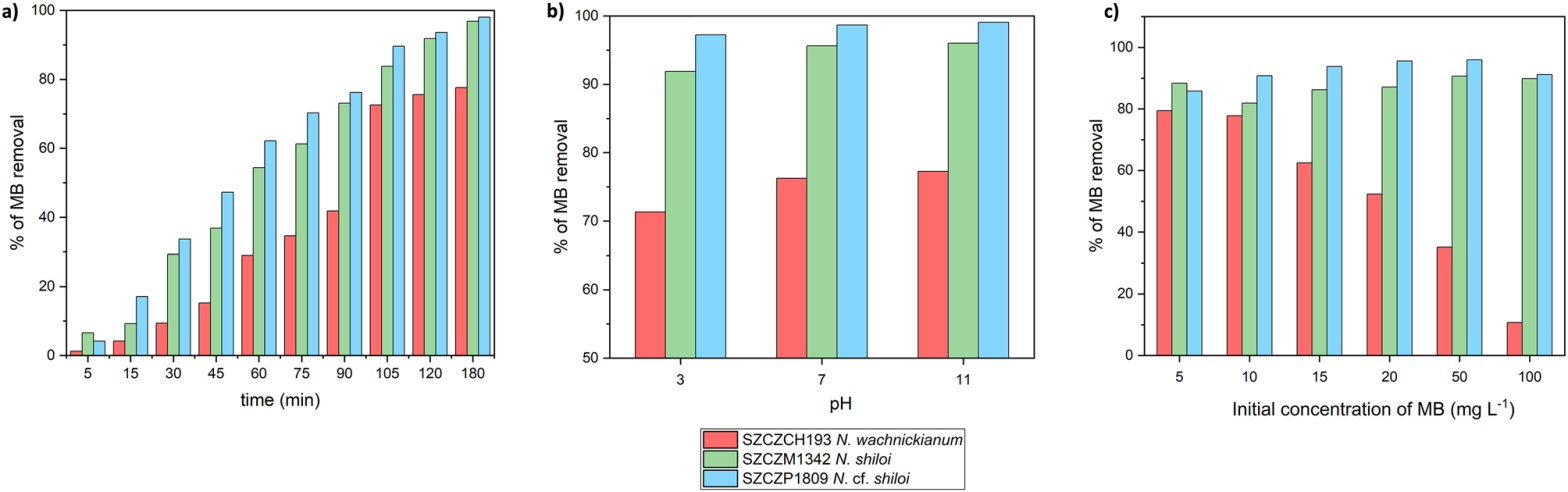
Effect of (**a**) time; (**b**) different pH and (**c**) different initial MB concentrations on the efficiency of MB adsorption onto pure biosilica of SZCZCH193 *N*. *wachnickianum*, SZCZM1342 *N. shiloi*, SZCZP1809 *N.* cf. *shiloi*, (initial concentration of MB—(**a**, **b**) 14.31 mg L^−1^; (**c**) 5–100 mg L^−1^; adsorbent dosage—21.40 ± 5.0 mg; pH—(**a**–**c**) 7, (**b**) 3, 7, 10; temperature—20 °C; time—(**a**) 5–180 min; (**b**, **c**) 120 min).

The pure porous diatomaceous biosilica of the three strains showed a positive response in the discoloration of MB in aqueous solution. Spectroscopy showed a sharp decrease in the MB peak with exposure time (Figure S3a-c), which confirmed the removal of the dye. The adsorption of MB onto the biosilica from SZCZCH193 *N*. *wachnickianum* strain progressed slowly with a sharp increase after 90 min of exposure until it reached equilibrium after 120 min. For frustules of SZCZCH193* N*. *wachnickianum*, the percentage of MB removal after 180 min was 77.6%. The adsorption of MB onto the frustules of SZCZM1342 *N. shiloi* and SZCZP1809 *N.* cf. *shiloi* gradually increased with time until the systems reached equilibrium after 120 min of exposure. The percentage of MB removal after 180 min was calculated as 96.8% for SZCZM1342 *N. shiloi* and 98.1% for SZCZP1809 *N.* cf. *shiloi* (Fig. 6a).

Furthermore, the effect of different pH of MB solutions was investigated with exposure of biosilica to acidic (pH = 3), neutral (pH = 7), and alkaline (pH = 11) aqueous solutions of MB as shown in Fig. 6b. In acidic solutions, the removal of MB was lower than in neutral and alkaline conditions: 71.33% for SZCZCH193 *N. wachnickianum*, 91.90% for SZCZM1342 *N. shiloi*, and 97.23% for SZCZP1809 *N.* cf. *shiloi*. An increase in pH resulted in higher removal efficiencies: up to 77.27% for SZCZCH193 *N. wachnickianum*, up to 96.03% for SZCZM1342 *N. shiloi*, and up to 99.08% for SZCZP1809 *N.* cf. *shiloi*.

The influence of different MB concentrations in the initial solution was also examined. Figure 6c shows that, for SZCZCH193 *N. wachnickianum*, an increase in MB concentration resulted in a significant decrease in the removal efficiency—from 79.48% at 5 mg L^−1^ to 10.69% at 100 mg L^−1^. Frustules of SZCZM1342 *N. shiloi*, exposed to a 100 mg L^−1^ solution of MB, showed an hyperbolic increase with concentrations almost of the same efficiency (89.82%) as at lower concentrations—88.35% at 5 mg L^−1^, 81.92% at 10 mg L^−1^, 86.26% at 15 mg L^−1^, 87.13% at 20 mg L^−1^, 90.69% at 50 mg L^−1^, and 89.82% of removal at 100 mg L^−1^. The most efficient strain in the removal of MB, SZCZP1809* N*. *wachnickianum*, showed a similar tendency—85.82% of removal at 5 mg L^−1^, 90.78% at 10 mg L^−1^, 93.77% at 15 mg L^−1^, 95.59% at 20 mg L^−1^, 95.95% at 50 mg L^−1^, and 91.15% at 100 mg L^−1^.

### Batch adsorption kinetics and isotherm studies

A pseudo-first order (Supplementary Figure S4a) model showed higher correlation coefficients for SZCZM1342 *N. shiloi* and SZCZP1809 *N.* cf. *shiloi* than Elovich (Supplementary Figure S4b) and pseudo-second order (Supplementary Figure S4c) kinetic models and the normalized standard deviation value for pseudo-first order was the lowest. For SZCZCH193* N*. *wachnickianum*, the correlation coefficients for pseudo-second and Elovich models were higher than for the pseudo-first order model, but not close to unity and with very small constant values. The standard deviation value was higher than 50% for all three models (Table 2).

**Table 2:** Adsorption kinetic and diffusion model constants for MB removal using pure diatomaceous mesoporous biosilica.

Kinetic model	Pseudo-first order				Pseudo-second order				Elovich				
	q_1_, mg g^−1^	k_1_, min^−1^	R^2^	Δq, %	q_2_, mg g^−1^	k_2_, g (mg min)^−1^	R^2^	Δq, %	α, g (mg min)^−1^	β, mg g^−1^	R^2^	Δq, %	
SZCZCH193	19.902	0.002	0.887	52.2	39.671	2.71e^−5^	0.894	65.4	0.042	0.051	0.893	64.7	
SZCZM1342	9.445	0.991	0.979	26.9	14.787	4.09e^−4^	0.964	28.2	0.094	0.178	0.969	29.6	
SZCZP1809	8.458	0.987	0.989	24.1	12.283	7.74e^−4^	0.973	28.9	0.130	0.237	0.976	32.3	

Diffusion model	Intra-particle (Weber-Morris)				Bangham’s pore diffusion								
	K_wm_, mg (g min ^0.5^)^−1^	B, mg g^−1^	R^2^	Δq, %	Δβ	K_β_	R^2^	Δq, %					
SZCZCH193	0.377	1e^−16^	0.730	52.2	1.374	0.004	0.972	18.2					
SZCZM1342	0.535	1e^−14^	0.901	26.9	1.025	0.037	0.964	13.9					
SZCZP1809	0.575	1e^−14^	0.930	24.1	1.073	0.035	0.985	19.2					

Isotherm	Langmuir				Freundlich				Sips				
	Q^0^_max_, mg g^−1^	K_L_, L mg^−1^	R^2^	Δq, %	K_F_, (mg g^−1^) (mg L^−1^)^−n^	n	R^2^	Δq, %	q_m_, mg g^−1^	K_S,_ [(mg L^−1^)^−1/n^]	n	R^2^	Δq, %
SZCZCH193	6.85	0.31	0.965	11.9	2.37	0.27	0.953	19.6	8.39	0.16	0.65	0.992	7.2
SZCZM1342	16.18	1.35	0.959	48.9	1.88	1.10	0.965	36.3	19.02	0.26	2.26	0.998	6.5
SZCZP1809	27.09	0.24	0.879	> 100	4.94	0.67	0.820	> 100	15.17	0.75	2.87	0.999	20.9

For the three tested strains, the Boyd’s linear graph was a straight line passing through the origin (see Supplementary Figure S4e), and the test showed high correlation coefficients (R^2^ = 0.962, 0.966 and 0.995 for SZCZCH193* N*. *wachnickianum*, SZCZM1342 *N. shiloi*, and SZCZP1809 *N.* cf. *shiloi* respectively) and low standard deviation values for SZCZM1342 *N. shiloi* (14.5%) and SZCZP1809 *N*. cf. *shiloi* (35.2%), but higher than 50% for SZCZCH193 *N. wachnickianum*.

To understand the rate-controlling step of the adsorption, Weber-Morris Intra-Particle Diffusion and Bangham’s Pore Diffusion models were used (Supplementary Figure S4d, f). The highest and closest to unity coefficient for SZCZCH193* N*. *wachnickianum*, SZCZM1342 *N. shiloi*, and SZCZP1809 *N.* cf. *shiloi* were calculated for the Bangham’s Pore Diffusion model. The standard deviation values for Bangham’s Pore Diffusion model for all three strains were lower than 20% (Table 2).

According to the isotherms models’ constants and coefficients of correlation, the Sips Isotherm model is the most suitable for SZCZCH193* N*. *wachnickianum*, SZCZM1342 *N. shiloi*, and SZCZP1809 *N.* cf. *shiloi* (Table 2, Supplementary Figure S5a–c).

## Discussion

Several studies reported specific growth rate and biomass productivities for *Nanofrustulum shiloi* strains as 0.099 d^−1^^38^, 0.305 d^−1^^39^, 0.457 d^−1^^40^, which correspond to 12.8 mg L^−1^ d^−1^^38^, 31.29 mg L^−1^ d^−1^^40^, respectively. In the present study, the specific growth rate and biomass productivity of SZCZM1342 *N. shiloi* and SZCZP1809 *N.* cf. *shiloi* were observed as 0.270 d^−1^, 0.513 d^−1^, which correspond to 26.12 mg L^−1^ d^−1^, 39.37 mg L^−1^ d^−1^, respectively. Thus, SZCZP1809 could be considered the most productive strain of *N. shiloi*. To the best of our knowledge no work has been done on the growth investigation of *Nanofrustulum wachnickianum*. A significant increase of biomass yield at lower temperatures for SZCZP1809 *N.* cf. *shiloi* and higher temperature for SZCZCH193 *N. wachnickianum* could be explained by the origin of the strains: SZCZP1809 was collected from the Atlantic Ocean, Sea Point, Cape Town, South Africa, where the average sea water temperature amounts to 17 °C, and SZCZCH193 *N. wachnickianum*—from the Gulf of Mexico, Marquesas Keys, Florida, USA (an average sea water temperature of 28 °C). Silica is a major limiting nutrient for diatom growth because of their need to build silicon-based frustules. The strains of *Nanofrustulum* spp. showed that biomass yield can be significantly enhanced with an increased silicon source up to almost 1 g L^−1^ DW, which correlated with previous studies^41^.

Pictures from scanning electron microscopy revealed that the valve faces of strains SZCZP1809 *N.* cf. *shiloi* and SZCZM1342 *N. shiloi* contain more than one row of areolae whereas the valves of SZCZCH193 *N. wachnickianum* contain only one row. The differences in areolation of the valve face make frustules of SZCZM1342 *N. shiloi* and SZCZP1809 *N.* cf*. shiloi* more porous than those of SZCZCH193 *N. wachnickianum.* Li et al.^42^ described a new species of *N. wachnickianum* and observed that this species could be differentiated from *N. shiloi* by the number of areolae on the valve face. In the case of MB removal, the pore size and number are crucial. Thus, SZCZCH193 *N. wachnickianum* has less potential as an adsorbing agent of MB due to a lower number of areolae, unlike SZCZM1342 *N. shiloi* and SZCZP1809 *N.* cf*. shiloi*. The areolae (width 80 nm and higher) of *Nanofrustulum* spp. strains could be considered macropores^43^. However, the N_2_ adsorption/desorption revealed presence of micro and mesopores, with average diameter 4.217 nm (SZCZCH193 *N. wachnickianum*), 2.073 nm (SZCZM1342 *N. shiloi*), and 1.971 nm (SZCZP1809 *N.* cf*. shiloi*). Previous studies reported the similar values of average pore diameter of diatomaceous frustules: 3.93 nm for *Pseudostraurosira trainorii*^44^, 3–10 nm for *Thalassiosira punctigera* and 3.6–3.7 nm for *T. weissflogii*^45^, and 4.61 nm for *Navicula australoshetlandica*^46^. Additionally, the porous nature of material could be described by the shape of the N_2_ adsorption/desorption isotherm. According to IUPAC classification the isotherms of *Nanofrustulum* spp. biosilica follow the Type I (microporous nature) and Type II (macroporous material), with combination of Type H3 and H4 hysteresis loop, where Type H3 shows presence of the macropores network and Type H4 indicates the existence of slit-like micropores in sample^43^.

Specific surface area of different strains of *NanofrusItulum* spp. differs slightly. Previous reports showed that different diatomaceous frustules have diverse S_BET_ value: from 2 m^2^ g^−1^ for *Skeletonema* sp.^47^ and 30 m^2^ g^−1^ for *P. trainorii*^44^, to 401.45 m^2^ g^−1^ for *N. australoshetlandica*^46^. However, it is important to note, that cleaning method could influence enormously in surface area: Gholami et al.^29^ demonstrated the possibility to increase SBET for *Cyclotella* sp. frustules from 14.71 m^2^ g^−1^ to 132.67 m^2^ g^−1^ with change of cleaning method from traditional to Sono-Fenton.

The appearance of peaks at 430, 495, and 676 nm in UV–vis spectra of sonicated biomass, according to interpretations in several reports^48,49^, confirmed the presence of chlorophyll and carotenoids. A peak at 270 nm could indicate the presence of polysaccharides as reported by Trabelsi et al.^50^. Sonicated, purified frustules showed only one peak at 230 nm, revealing the presence of silica in the samples^51,52^.

First stage of thermal analysis is connected with water desorption, second stage—could be connected to oxidative degradation of organic compounds (i.e. lipids), while third stage revealed degradation of inorganic matter (i.e. magnesium and sodium salts)^53–55^. The highest mass loss (almost 75%) of the sample during the thermal analysis (TGA/DTA) was observed for the sample of SZCZM1342 *N. shiloi*. In comparison for samples SZCZCH193 *N. wachnickianum* and SZCZP1809 *N.* cf. *shiloi* the overall mass loss was 45.8% and 43.8%. This could be explained by presence of high amount of organic matter in sample SZCM1342 *N. shiloi*, considering that this sample had slightly brown color in comparison to other samples (see Supplementary Figure S6).

The EDS study confirmed the presence of silica and oxygen in the frustules. The atomic ratio of O:Si was calculated as 1.69:1, 2.39:1, and 2.27:1 for SZCZCH193, SZCZM1342, and SZCZP1809, respectively, which confirmed that diatom frustules are made from silica mostly in the form of SiO_2_ nH_2_O—very similar to opal^56^. The distinct peak of carbon (C) could be explained by the presence of the remaining organic compounds surrounding the frustules and chitin template within the diatomaceous silica^57^. The ooccurrence of copper (Cu) is the result of using copper TEM grids.

X-ray diffraction analysis for all strains showed the broad peak indicating the presence of amorphous from of the silica. Thus, it can be concluded that studied samples mostly comprised from amorphous silica. For SZCZCH193 N. wachnickianum intense signals are most likely originating from quartz and cristobalite^58^. Alternatively, these signals could indicate the inclusions of magnesium inorganic salts^53^. For SZCZP1809 *N.* cf. *shiloi* the low intensity signals may come from the inclusions of alpha-quartz.

The calculated Tauc plot energy bandgaps (4.40 eV for SZCZCH193 *N*. *wachnickianum*, 4.05 eV for SZCZM1342 *N*. *shiloi*, and 4.10 eV for SZCZP1809 *N*. cf. *shiloi*) showed that pure porous biosilica has the properties to be an ultrawide semiconductor^59^. Analysis for amorphous silica^60^ revealed a band gap of 3.35 eV. A previous study confirmed that amorphous and crystalline silica has a lower band gap than a porous one^61^ and the difference in values for strains could be explained by their differences in pore sizes.

In order to identify the potential functional groups on the surface of the frustules, ATR-FTIR analysis was performed. According to several reports^12,62^, the appearance of strong bands at 1150, 1050, 805 and 442 cm^−1^ presented asymmetrical stretching, symmetrical stretching, and bending vibrations of Si–O–Si groups, respectively. There are strong bands recognised by several studies^12,62,63^ as C–H stretching at 2981, 2922 (CH_3_), 2851 (CH_2_), and 854 cm^−1^ (Si–(CH_3_)_2_), and C=O stretching at 1748 cm^−1^. Four strong peaks at 1630, 1540, 1450, and 1395 cm^−1^ showed the bending vibrations of amino groups, corresponding to a previous study^64^. According to Otzen^65^, these peaks indicate the presence of organic compounds surrounding the biosilica. The band at 3660 cm^−1^ is related to a stretching vibration of O–H from SiOH^62^, moreover, the broad band from 3500 to 3000 cm^−1^ is related to molecular water^66^.

The surface charge of the particles determines the ability of the particles to aggregate. The value of zeta potential is dependent on the properties of the given particles as well as from the pH and ionic strength of the solution. Particles with zeta potential close to zero will aggregate. In turn, stable and non-aggregating systems are characterized by absolute zeta potential values greater than +/− 25 − + /− 30 mV^67,68^. The results of zeta potential measurments for pure biosilica samples indicate that at pH greater than 4 aggregation is not occurring and suspensions are stable. The respective results can be confirmed by the photos taken of diatom solutions at different pH (2.0, 3.0, > 4.0) (see Supplementary Figure S7a-c). In the lowest pH a significant aggregation and sample precipitation can be observed. At pH around 3.0 the aggregation is still visible but in lower extend. At pH higher than 4.0 no visible aggregation occurs, the suspension is stable. It is noteworthy to mention, that obtained results differ from the results obtained for pure synthetically prepared silica described by Xu et al.^69^. The different shape of zeta potential plot of examined samples in comparison to Peng Zu’s can be explained by the presence of carboxyl (COOH) and amine (NH_2_) groups on surface of the biosilica. The presence of respective functional groups was confirmed by FTIR analysis. Moreover, TA/DTA analysis also revealed the presence of high amount of organic matter on the surface of the biosilica. The difference is also notable between the samples of biosilica, for instance, for SZCM1342 *N. shiloi* the positive charge of the surface was observed. The respective difference more likely is due to higher amount of organic matter on the surface of SZCZM1342 *N. shiloi* sample, (i.e. proteins).

The point zero charge (PZC) allows us to find the pH at which the charge at the surface is neutral, therefore in pH less than pH_pzc_ the surface is charged positively, and at pH higher than pH_pzc_ the charge of the surface is negative^70^. Pure biosilica showed a PZC value of pH_pzc_ = 6.0 (SZCZCH193 *N*. *wachnickianum*), and pH_pzc_ = 5.3 (SZCZM1342 *N*. *shiloi*, SZCZP1809 *N.* cf. *shiloi*), which corresponds with several studies of diatomaceous earth: the residual (RDE) and pure (PDE) from Brazil (pH_pzc_ = 6.75 and pH_pzc_ = 6.59 respectively)^70^, from the mine El Pino (pH_pz_c = 5.0)^71^, from East Jordan (pH_pzc_ = 5.4)^72^, from Egypt (pH_pzc_ = 6.21)^73^. The results from the PZC study of pure diatomaceous biosilica confirm the presence of O–H groups on the surface and suggest that these hydroxyl groups can gain or lose a proton by changing the pH. Therefore, in acidic media (pH < pH_pzc_), the Si–OH group of biosilica gains a proton and produces Si–OH_2_^+^, and in basic media (pH > pHpzc) the Si–OH group loses a proton and produces Si–O^−^^74^.

The percentage of MB removal from aqueous solution in all experiments for SZCZCH193 *N. wachnickianum* was lower than for the two remaining strains of *N. shiloi*, these differences could be explained by differences in morphology, pore density, specific surface area, and pore diameter between the *Nanofrustulum* spp. strains. Nevertheless diatomaceous biosilica showed good removal efficiency compared with other bioadsorbents: 80% for pine tree needles after 240 min of incubation and at pH = 9.2^75^, 53% for bacteria^21^, up to 90% for fungi *Phellinus adamantinus*^23^, up to 90% for sugarcane bagasse^25^. Cleaned frustules of SZCZP1809 *N.* cf. *shiloi* demonstrated an efficiency close to activated carbon prepared from rice husk—98.43% at a flow rate of 1.0 ml min^−1^^76^. Natural diatomite, a more explored adsorbent than cleaned diatom biosilica (frustules), showed a similar removal efficiency—95.2% at pH = 10^73^, 90.75% and 100 mg L^−1^ MB concentration^77^, 96.5% for modified diatomite^78^, and 100% at 50 mg L^−1^ of MB^79^.

Abdelrahman et al.^80^ noticed a decrease in the removal of MB under increased concentrations for metal–silica amorphous adsorbents, similar to the removal observed for SZCZCH193 *N. wachnickianum*. However, the hyperbolic increase with concentration for SZCZM1342 *N. shiloi* and SZCZP1809 *N.* cf. *shiloi* was noticed as for MB absorbance onto fava bean peel^81^.

Even though the frustules of *Nanofrustulum* spp. showed a high percentage of MB removal, comparable to the efficiency of well-known adsorbents such as natural diatomite and activated carbon, the MB adsorption capacity of *Nanofrustulum* spp. frustules was reported almost 10 times less than diatomite^32^, algae *Gellidium* sp.^22^, little less than amorphous silica^82^, brown algae biomass^83^, and almost the same as some zeolites^26^ and dead biomass of *Aspergillus niger*^84^ (see Supplementary Table S2). Pre-treated frustules of *Pinnularia* showed a higher adsorption capacity^28^ than frustules of *Nanofrustulum* spp., which could be explained by morphology and specific surface area of *Pinnularia* and *Nanofrustulum*, as well as by the cleaning method.

The pH of adsorbate solution is considered to be one of the most important parameters in water adsorption processes^74^. The pKa of MB is reported to be 3.8, thus at pH less than 3.8 the surface of MB molecule is not charged, and at pH higher than 3.8—positively charged^85^. Therefore, in pH less than pH_pzc_ of biosilica, the main interaction between MB and frustules are hydrogen bonds and the adsorption process is slower because of repulsive forces between positive sites on the diatomaceous biosilica surface and the cationic dye. In pH higher than pH_pzc_ of biosilica, the positively charged MB and negatively charge frustules interact electrostatically, and the adsorption process is stronger than in acidic media^73^. In our study, an increase in pH resulted in increased removal up to 99.08% for SZCZP1809 *N.* cf. *shiloi*. Similar results reported for diatomite from China^79^, diatomaceous earth from Egypt^80^, East Jordan^72^ and Brazil^71^, palygorskite^74^. To achieve desirable pH, trace amounts of 1 M HCl and 1 M NaOH solutions were added and could negatively affect the adsorption as an ionic species (Na^+^ and Cl^−^), because in presence of inorganic salts the adsorbent surface becomes not easily accessible for MB. However, there were no influence of these ionic species on the dye uptake observed, presumably due to their low concentrations (less than 0.005 M) in dye solution^86^.

Moreover, several kinetic, diffusion, and equilibrium models were applied for better understanding of the possible MB sorption mechanism. The kinetic curve fitted the pseudo-first order model better (with a higher correlation coefficient and lower standard deviation value), therefore we can conclude that the adsorption behaviour of MB on biosilica predominantly followed the pseudo-first order kinetic model: the overall rate of adsorption process was controlled by physisorption, meaning that the molecular interaction between MB and biosilica is governed by van der Waals froces^87^. In order to determine whether the main resistance to mass transfer was in the thin film (boundary layer) surrounding the adsorbent particle, or in the resistance to diffusion inside the particles, Boyd’s model was applied. The straight line passing through the origin indicated that the MB adsorption rate is governed by diffusion inside the particles^88^. The high correlation coefficients for Bangham’s pore diffusion model indicated that the diffusion of MB molecules onto pores inside biosilica is a rate-controlling step in mass transfer for adsorption process^89^. The adsorption isotherms of SZCZM1342 and SZCZP1809 were characterized as slightly LS-shaped and the isotherm of SZCZCH193 as L-shaped^90^, which suggests that the studied diatomaceous biosilica exhibit a high adsorption affinity towards MB dye. Isotherms for *Nanofrustulum* spp. showed a higher affinity to the Sips equation due to high correlation coefficients and low standard deviation values. Based on that, we can conclude that the mechanism of MB sorption onto biosilica predominantly follows the Sips model, which combines the Freundlich and Langmuir isotherms and describes monolayer MB formation onto homogenous and heterogenous sites on biosilica surface^90^.

The present research introduced a novel environmentally friendly adsorbent of basic dyes from wastewaters—the porous biosilica originated from marine diatom *Nanofrustulum*. The diatom genus could be considered the most productive microalgae with the highest biomass productivity. The biomass could be further used for extraction of bio-active molecules, e.g. fucoxanthin, known by its antioxidant activity, polyunsaturated fatty acids, with anti-inflammatory properties, and neutral lipids for biodiesel production, while unused diatomaceous biosilica can be purified and efficiently applied in wastewater treatment, due to its porous architecture, the negative surface charge, and relatively high the specific surface area. Several researchers reported the ability of diatomaceous silica to remove heavy metals from aqueous solutions, although the present paper for the first time introduced biosilica as a novel efficient adsorbent of basic dyes from wastewaters. In the future, more intensive research should focus on evaluation of biorefinery potential of these three species, with special focus on co-production of fucoxanthin, fatty acid, and biosilica in economically desirable and eco-friendly way, and adsorption capacity to remove different types of organic pollutants in presence of inorganic salts, which could decrease dye uptake.

## Materials and methods

### Chemicals

Methylene blue (> 99%, MW 319.89 Da) was purchased from Aqua-Med® (Łódź, Poland). Thiamine hydrochloride (99%, MW 337.27 Da), biotin (> 99%, MW 244.31 Da), vitamin B12 (> 98%, MW 1355.37 Da) were supplied by Sigma-Aldrich (St. Louis, MO, USA). Hydrogen peroxide (30%, MW 34.01 Da), sodium nitrate (> 99%, MW 84.99 Da), sodium dihydrogen phosphate monohydrate (> 99%, MW 137.99 Da), sodium molybdate dihydrate (> 99%, MW 241.95 Da), manganese (II) chloride tetrahydrate (> 99%, MW 197.91 Da), and cobalt (II) chloride hexahydrate (> 99%, DW 237.93 Da) were obtained from Chempur® (Piekary Śląskie, Poland). Zinc sulfate heptahydrate (> 99%, MW 287.54 Da), iron (III) chloride hexahydrate (> 99%, MW 270.32 Da), EDTA disodium dihydrate (> 99%, MW 372.24 Da), and copper (II) sulfate pentahydrate (> 99%, MW 249.68 Da) were purchased from Scharlab (Barcelona, Spain). Nonahydrate sodium metasilicate (44–47.5% total solids, MW 284.19 Da) was supplied by Acros Organics, ThermoFisher Scientific (Waltham, MA, USA). Sodium hydroxide, hydrohloric acid and standard buffered solutions pH 2.0, 7.0 and 10.0 were purchased from Sigma-Aldrich. Deionized water was obtained by using a Milli-Q® purification system (Millipore Co., Bedford, MA, USA).

### Batch culturing optimization

Three diatom strains belonging to the genus *Nanofrustulum* were selected as follows: SZCZCH193 *N. wachnickianum* Chunlian Li, A.Witkowski & M.P.Ashworth; SZCZM1342 *N. shiloi* (J.J.Lee, Reimer & McEnery) Round, Hallsteinsen & Paasche^42^; and strain SZCZP1809 morphologically identified by P. Dąbek as *N.* cf. *shiloi* (sampled from Sea Point, Cape Town, South Africa) were obtained from the Szczecin Diatom Culture Collection (SZCZ), University of Szczecin, Institute of Marine and Environmental Sciences, Poland. Monoclonal cultures were maintained in standard 35‰ Guillard′s artificial seawater f/2 medium^91^ (880 µM L^−1^ NaNO_3_, 36 µM L^−1^ NaH_2_PO_4_ H_2_O, 106 µM L^−1^ Na_2_SiO_3_ 9H_2_O, trace metal: 0.08 µM L^−1^ ZnSO_4_ 7H_2_O, 0.9 µM L^−1^ MnSO_4_ H_2_O, 0.03 L^−1^ µM Na_2_MoO_4_ 2H_2_O, 0.05 µM L^−1^ CoCl_2_ 6H_2_O, 0.04 µM L^−1^ CuCl_2_ 2H_2_O, 11.7 µM L^−1^ FeCl_3_ 6H_2_O, 11.7 µM L^−1^ Na_2_EDTA 2H_2_O, vitamin B12, biotin and thiamine) at constant temperature (20 °C) and illumination (100 µmol s^−1^ m^−2^ white light) under a 12:12 day/night light cycle in a plant growth chamber (FITO1400i, Biogenet, Poland). The growth rates were determined by dry biomass weight harvested by centrifugation at 3000 rpm speed and heat drying for 3 days at 50 °C. The regression curve for each strain was built and the specific growth rate (µ) was calculated using the following Eq. (1) ^92^:

1 $$B_{t} = B_{0} \times e^{\mu t}$$

where *B*_*t*_ is the biomass concentration at any time (*t*) and *B*_0_ is the initial biomass concentration. The diatom strains were observed under an Olympus CKX41 inverted microscope (Olympus- Shinjuku, Tokyo, Japan) at 400 × magnification.

The influence of nutrient enrichment (5, 10, 15, 20 times higher concentration of NO_3_^−^, PO_4_^3−^, SiO_3_^2−^), salinity (15, 20, and 45%), temperature (15 and 30 °C), and illumination intensity (10, 50, and 150 μmol photons m^−2^ s^−1^) on biomass accumulation in the late exponential phase was studied for each strain.

### Characterization of the diatomaceous biosilica

Diatoms were harvested in the late exponential phase, centrifuged at 3000 rpm for 15 min, and pellets were purified by 30% H_2_O_2_ solution at 110 °C for 2 days following a thorough cleaning with ddH_2_O. The colorless, cleaned silica was dried at 50 °C for 3 days and later used for characterization experiments. The morphology of the clean diatom frustules was imaged by scanning electron microscopy (SEM) using a Hitachi SU8000 (Hitachi, Tokyo, Japan). For the SEM study, 40 µL of frustules were dried on a Nuclepore™ 5.0 µm Track-Etch Membrane (Whatman™, Cytiva, Germany) at room temperature, later mounted on a M4 cylinder SEM holder, and sputtered with a 10 nm thick gold layer. For the elemental analysis of biosilica, a drop of frustules was placed on a carbon-coated copper grid (Sigma-Aldrich, USA), and the analysis was carried out by a Hitachi STEM S5500 equipped with an EDS detector (Hitachi, Tokyo, Japan). The measurements were performed with an accelerating energy of 30.0 kV and analyzed using NSS ThermoScientific software.

After sonication of the biomass and the cleaned frustules (using Hielscher UP100H ultrasonic processor (Teltow, Germany) for 20 min at 60% amplitude), the suspensions were subjected to the UV–Vis DR 6000 spectrophotometer (HACH-Lange) for optical measurements in the wavelength range of 200–900 nm in 10 mm cuvettes against ddH_2_O (blank). For absorbances higher than 2, the solution was diluted with ddH_2_O, the dilution factors were considered in the presented graphs.

FTIR spectra of dried biosilica were obtained in the mid-infrared range (4000–400 cm^−1^) with the utilization of attenuated total reflection (ATR) mode on an Alpha FTIR spectrometer (Bruker Daltonics, Bremen, Germany).

The low-temperature nitrogen adsorption/desorption isotherms were recorded on Quantachrome Autosorb iQ at 77.35 K.

Thermogravimetric analysis (TA/TGA) of biosilica samples was performed with TA Instruments type SDT 2960 (Artisan Technology, Champaign, IL, USA) using 0–1100 °C temperature range, 100 mL min^−1^ air flow rate and 10 °C min^−1^ heating rate.

Zeta potential measurements were carried out with Malvern Zetasizer NanoZS (Malvern) using DTS1070 cuvette (Malvern). The analysis was performed in the automatic selection mode of voltage and number of runs. Each measurement was repeated three times. Zeta potential was measured in 2.0–12.0 pH range. To maintain the pH 0.1 M NaOH and 0.1 M HCl solutions were used. FiveEasy Plus pH-meter (Mettler Toledo) with a combined electrode with glass membrane and Ag/AgCl reference system (Mettler Toledo) was applied to measure pH of suspensions. The pH-meter was calibrated using standard buffered solutions with pH of 4.0, 7.0, 10.0 before carrying out measurements.

The X-ray diffraction (XRD) spectra were recorded with an X’Pert Pro Analytical X-ray diffractiometer (Phillips, Würzburg, Germany) with Cu-Kα radiation (λ = 0.1541 nm, 40 kV, 30 mA); 1 mL of dried on glass slide sample was scanned in the 2θ range between 5° and 120° with step sizes of 0.0167.

The salt addition method was used to find the PZC of dried biosilica. An aliquot of 20 ml of 0.1 M NaCl was collected in 6 Erlenmeyer flasks and a 2–10 pH range was set by adding 1 M HCl and 1 M NaOH solutions using a pH meter (Voltcraft PH-100ATC). Then in each flask 20 mg of the dried biosilica were added and shaken at speed 100 rpm on an orbital shaker at temperature 23 °C for 24 h. After equilibrium, the contents were filtered, and the pH values of filtrates were recorded. The PZC value was determined by plotting the graph of initial pH against the change in pH.

### Methylene blue (MB) batch sorption

The cleaned frustules (20 ± 0.5 mg DW) were exposed to 10.0 ml of 14.0 mg L^−1^ MB in a 15 ml Falcon tube. The mixture (pH = 7) was mechanically stirred at 3000 rpm at 23 °C for 3 h. The removal of dye was recorded by a UV–Vis DR 6000 (HACH-Lange) spectrophotometer in the wavelength range from 500 to 800 nm at different time points: 5, 15, 30, 45, 60, 75, 90, 105, 120, and 180 min.

Similarly, isotherm studies were performed by measuring 20 ± 0.5 mg of cleaned frustules into 15 ml Falcon tubes containing varying initial concentrations (5, 10, 15, 20, 50, and 100 mg L^−1^) of MB. The mixture was stirred at 3000 rpm under 23 °C for 120 min, which is necessary to attain equilibrium. The effect of different pH of the initial MB solution (14.0 mg L^−1^) was recorded for pH = 3, 7, and 11 after 120 min of exposure. The initial pH of the solution was adjusted with 1 M HCl and 1 M NaOH solutions. The absorbance value at 665 nm was used for further calculations.

The quantity of adsorbed MB by biosilica was calculated as follows:

2 $$q_{t} = \frac{{\left( {C_{0} - C_{t} } \right) \times V}}{m}$$

where *q*_*t*_ is the MB adsorbed on the biosilica (mg g^−1^) at a given time (*t*); *C*_*0*_ and *C*_*t*_ are the concentrations of the MB at the start and at the given timepoint (mg L^−1^), respectively; *V* is the solution volume (L); *m* is the biosilica dosage (g).

The percentage of MB removal (%) was calculated by Eq. (3):

3 $$removal \% = \frac{{\left( {C_{0} - C_{t} } \right) \times 100\% }}{{C_{0} }}$$

where *C*_0_ and *C*_*t*_ are the concentrations of the MB at the start and at the given time (*t*), respectively (mg L^−1^).

To understand the possible mechanisms and rate controlling steps of adsorption, several kinetic, diffusion and isotherm models were applied (see Supplementary Table S3).

Furthermore, the applicability of the kinetic and isotherm models was validated by the normalized standard deviation, *Δq* (%), Eq. (4)

4 $${{\Delta }}q = 100 \times \sqrt {\frac{{\sum \left[ {\frac{{q_{{exp}} - q_{{cal}} }}{{q_{{exp}} }}} \right]^{2} }}{{\left( {N - 1} \right)}}}$$

where *N* is the number of data points, *q*_*exp*_ and *q*_*cal*_ (mg g^−1^) are the experimental and calculated adsorption capacity value, respectively.

### Data analysis

The batch cultivation experiments were conducted in duplicate. The figures show mean values and standard errors. The significance of differences between different groups was analyzed using a one–way ANOVA analysis with Tukey's post-hoc test, the alpha level 0.05. The batch growth experiment figures, UV–Vis, FTIR spectra, batch adsorption spectra were plotted using MS Excel software. The EDS spectra were obtained using NSS ThermoScientific software. The zeta potential results take into account the Smoluchowski approximation. The analysis data for TA/DTA was proceed with the use of TA Universal Analysis software (TA Instruments, New Castle, DE, USA). The X-ray diffraction pattern analysis was obtained from XRD Malvern Panalytical software (version 1.5a, Almelo, The Netherlands). Modeling of adsorption were performed in OriginPro 2022 software.

## Supplementary Information

Supplementary Information.

## Data Availability

The authors confirm that the data supporting the findings of this study are available within the article and its Supplementary material. Raw data that supports the fundings of this study are available from the corresponding author, upon reasonable request.
