# Supplementary material for: A novel effective bio-originated methylene blue adsorbent: the porous biosilica from three marine diatom strains of Nanofrustulum spp. (Bacillariophyta)
Source: Sci Rep. 2023 Jun 6;13:9168. doi: 10.1038/s41598-023-36408-6 (PMC10244400; doi:10.1038/s41598-023-36408-6)
Supplement: Supplementary file 1 — Supplementary Information. [file 41598_2023_36408_MOESM1_ESM.docx]

**A novel effective bio-originated methylene blue adsorbent – the porous biosilica from three marine diatom strains of *Nanofrustulum* spp. (Bacillariophyta)**

Aleksandra Golubeva^1*^, Piya Roychoudhury^1^, Przemysław Dąbek^1^, Jagoda Pałczyńska^2^, Oleksandra Pryshchepa^3^, Piotr Piszczek^2^, Paweł Pomastowski^3^, Michał Gloc^4^, Renata Dobrucka^4,5^, Agnieszka Feliczak-Guzik^6^, Izabela Nowak^6^, Krzysztof J. Kurzydłowski^7^, Bogusław Buszewski^8,9^ and Andrzej Witkowski^1^

**Supplementary material**

**Supplementary Table S1.** Surface porous structure for SZCZCH193 *N*. *wachnickianum*, SZCZM1342 *N. shiloi*, SZCZP1809 *N.* cf. *shiloi* frustules.

| Strain | Specific Surface Area (m^2^ g^-1^) | Pore Volume (cm^3^ g^-1^) | Average Pore Diameter (nm) |
| --- | --- | --- | --- |
| SZCZCH193 *N. wachnickianum* | 25.319 | 0.267 | 4.217 |
| SZCZM1342 *N. shiloi* | 21.777 | 0.113 | 2.073 |
| SZCZP1809 *N*. cf. *shiloi* | 35.231 | 0.174 | 1.971 |

**Supplementary Table S2.** Adsorption capacity of different adsorbents

| Adsorbent | Adsorption capacity (mg g^-1^) | Reference |
| --- | --- | --- |
| Defatted *Scenedesmus* sp. biomass | 7.73 | [1] |
| Amorphous silica | 22.66 | [2] |
| Zeolite | 19.94 | [3] |
| Diatomite | 116.59 | [4] |
| Brown algae *Cystoseira barbatula* | 38.61 | [5] |
| Dead fungus *Aspergillus niger* | 18.54 | [6] |
| Algae *Gelidium* sp. | 171 | [7] |
| Pre-treated *Pinnularia* frustules | 26.2 | [8] |
| Diatomaceous mesoporous biosilica *Nanofrustulum* spp.  SZCZCH193 *N. wachnickianum*  SZCZM1342 *N. shiloi*  SZCZP1809 *N*. cf. *shiloi* | 8.39  19.02  15.17 |  |

**Supplementary Table S3.** Kinetic, diffusion and isotherm models used in this study

| Models | Equation |  | Description of parameters | References |
| --- | --- | --- | --- | --- |
| Kinetic study | | | | |
| *Pseudo-first order  (nonlinear)* | $q_{t}=q_{1}\left( 1-e^{-kt} \right)$ | (4) | *q_t_* (mg g^-1^) – amount adsorbed at given time (*t*, min)  *q_1_* (mg g^-1^) – adsorbent capacity at equilibrium  *k* (min^-1^) – Pseudo-first order constant rate  *q_2_* (mg g^-1^) – adsorbent capacity at equilibrium  *k_2_* (g (mg min)^-1^) – Pseudo-second order constant rate  *α* (mg (g min)^-1^) – initial adsorption rate  *β* (mg g^-1^) – desorption constant | [9] |
| *Pseudo-second order (nonlinear)* | $q_{t}=\frac{k_{2}q_{2}^{2}t}{1+k_{2}q_{2}t}$ | (5) |  | [10] |
| *Elovich (nonlinear)* | $q_{t}=\frac{1}{\beta}\ln(1+\alpha\beta t)$ | (6) |  | [11] |
| Diffusion study | | | | |
| *Intra-particle diffusion (nonlinear)* | $q_{t}=k_{wm}t^{0.5}+B$ | (7) | *q_t_* (mg g^-1^) – amount adsorbed at given time (*t*, min)  *K_wm_* (mg (g min^0.5^)^-1^)– Intra-particle diffusion rate constant  *B* (mg g^-1^) – intercept  *q_e_* (mg g^-1^) – amount adsorbed at equilibrium  *B_t_* – mathematical function of F  *F* – the fraction of metal ion adsorbed at any time (*t*)  *C_0_* (mg L^-1^) – initial concentration of MB  *m* (g) – mass of adsorbent g  *Δβ* and *K_β_* - Bangham сonstants  *V* (mL)– volume of solution | [12] |
| *Boyd (linear)* | $B_{t}=-0.4977-\ln(1-\frac{q_{t}}{q_{e}})$  *B_t_ vs. t plot* | (8) |  | [13] |
| *Bangham’s pore diffusion (linear)* | $\log\log\left( \frac{C_{0}}{C_{0}-mq_{t}} \right)=\log\left( \frac{mK_{\beta}}{2.303V} \right)+\Delta\beta\log t$  $\log\log\left( \frac{C_{0}}{C_{0}-mq_{t}} \right)$ *v.s* $\log t$ *plot* | (9) |  | [14] |
| Equilibrium study (isotherms) | | | | |
| *Langmuir (nonlinear)* | $q_{\boldsymbol{e}}=\frac{Q_{max}^{0}K_{L}C_{e}}{1+K_{L}C_{e}}$ | (10) | *q_e_* (mg g^-1^) – adsorption capacity at equilibrium  *C_e_* (mg L^-1^) – concentration of MB dye at equilibrium  *Q^0^_max_* (mg g^-1^) – maximum monolayer adsorption capacity of Langmuir  *K_L_* (L mg^-1^) – Langmuir constant  *K_F_* [(mg g^-1^) (mg L^-1^)^-n^] – Freundlich constant  *n* – dimensionless Freundlich intensity parameter  *q_m_* (mg g^-1^) – the Sips maximum adsorption capacity  *K_S_* [(mg L^-1^)^-1/n^] – the Sips equilibrium constant  *n* – the exponent of Sips where 0<1/n≤1 | [15] |
| *Freundlich (nonlinear)* | $q_{e}=K_{F}C_{e}^{n}$ | (11) |  | [16] |
| *Sips (nonlinear)* | $q_{t}=\frac{q_{m}\left( K_{S}C_{e} \right)^{n}}{1+\left( K_{S}C_{e} \right)^{n}}$ | (12) |  | [17] |

**
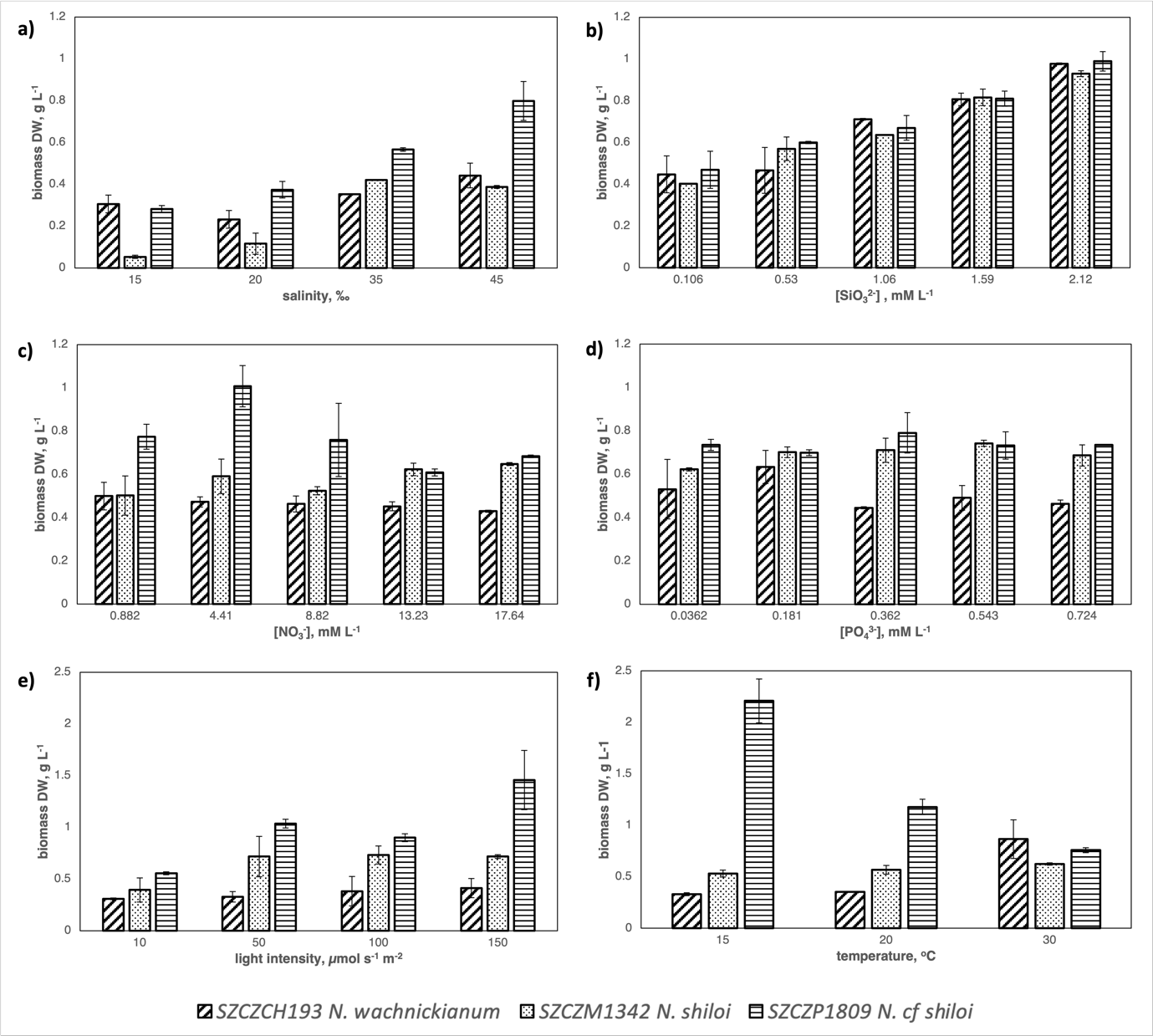
**

**Supplementary Figure S1.** Optimization of batch cultivation of SZCZCH193 *N*. *wachnickianum*, SZCZM1342 *N. shiloi*, SZCZP1809 *N.* cf. *shiloi*: the influence of salinity (a), silicate (b), nitrate (c), and phosphate (d) concentrations in f/2 medium, light intensity (e), and temperature (f) on biomass DW accumulation.


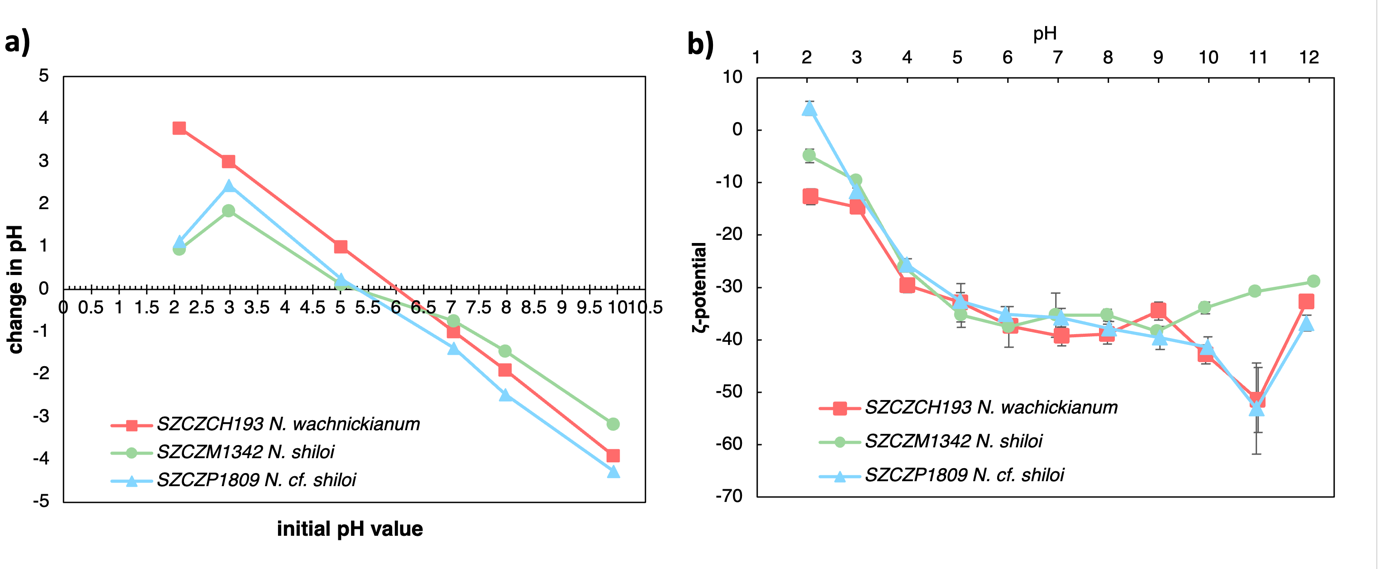


**Supplementary Figure S2.** (a) The point zero charge (PZC) and (b) the zeta potential of pure diatomaceous biosilica of SZCZCH193 *N*. *wachnickianum*, SZCZM1342 *N. shiloi*, SZCZP1809 *N.* cf. *shiloi*


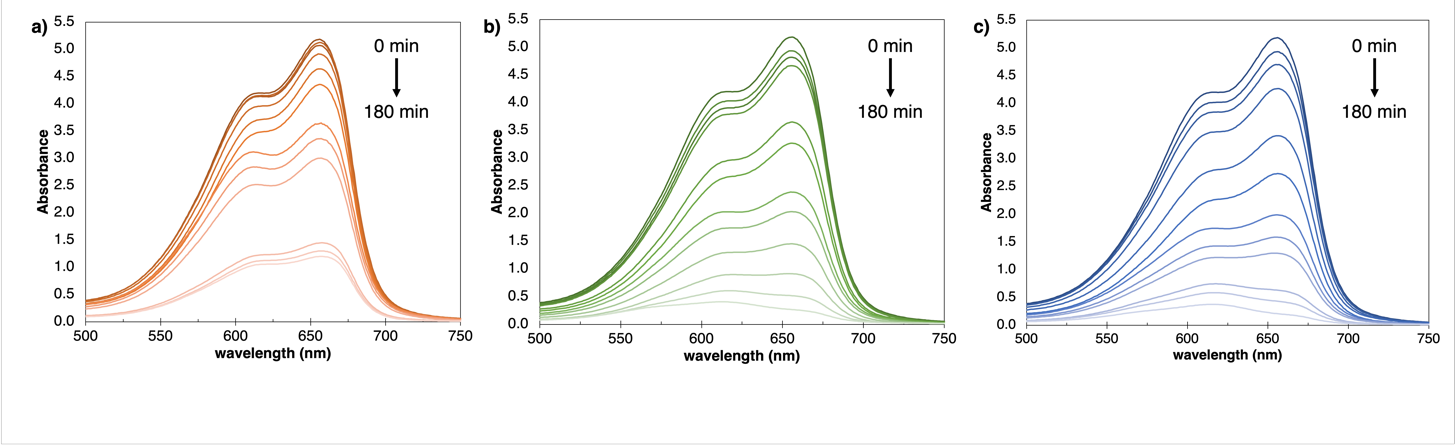


**Supplementary Figure S3.** The UV-vis spectra showing MB removal with time onto pure biosilica of (a) SZCZCH193 *N*. *wachnickianum*, (b) SZCZM1342 *N. shiloi*, (c) SZCZP1809 *N.* cf. *shiloi*


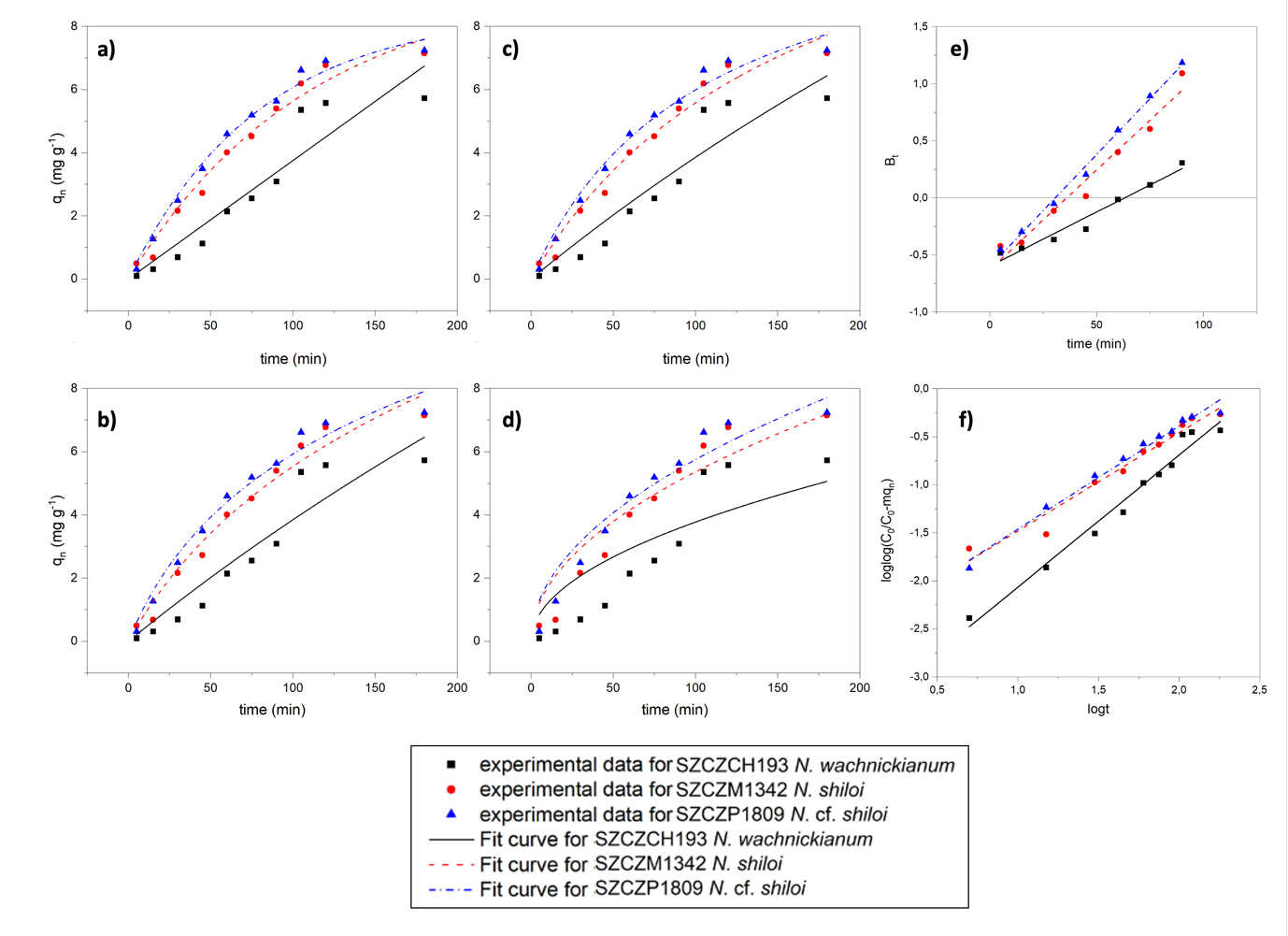


**Supplementary Figure S4.** Adsorption kinetic study: (a) pseudo-first order, (b) Elovich, and (c) pseudo-second order models, and diffusion study: (d) Intra-particle diffusion (Webber-Morris), (e) Boyd’s and (f) Bangham’s pore diffusion models (experimental data: black squares – SZCZCH193 *N*. *wachnickianum*, red circles – SZCZM1342 *N. shiloi*, blue triangles – SZCZP1809 *N.* cf. *shiloi;* calculated modeling data: black line for SZCZCH193 *N*. *wachnickianum*, red line for SZCZM1342 *N. shiloi*, blue line for SZCZP1809 *N.* cf. *shiloi*).


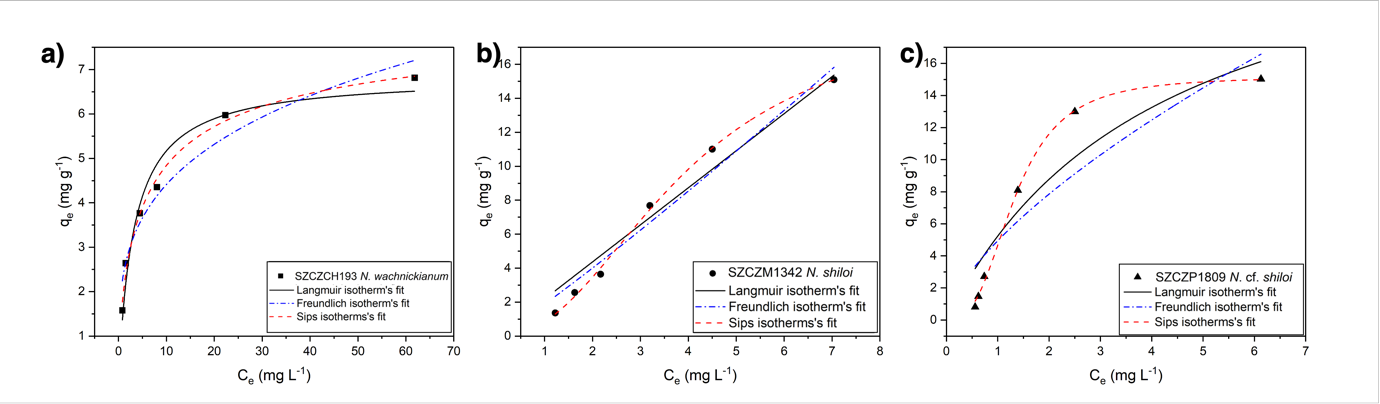


**Supplementary Figure S5.** Adsorption isotherms of MB onto pure biosilica of (a) SZCZCH193 *N*. *wachnickianum*, (b) SZCZM1342 *N. shiloi*, (с) SZCZP1809 *N.* cf. *shiloi*


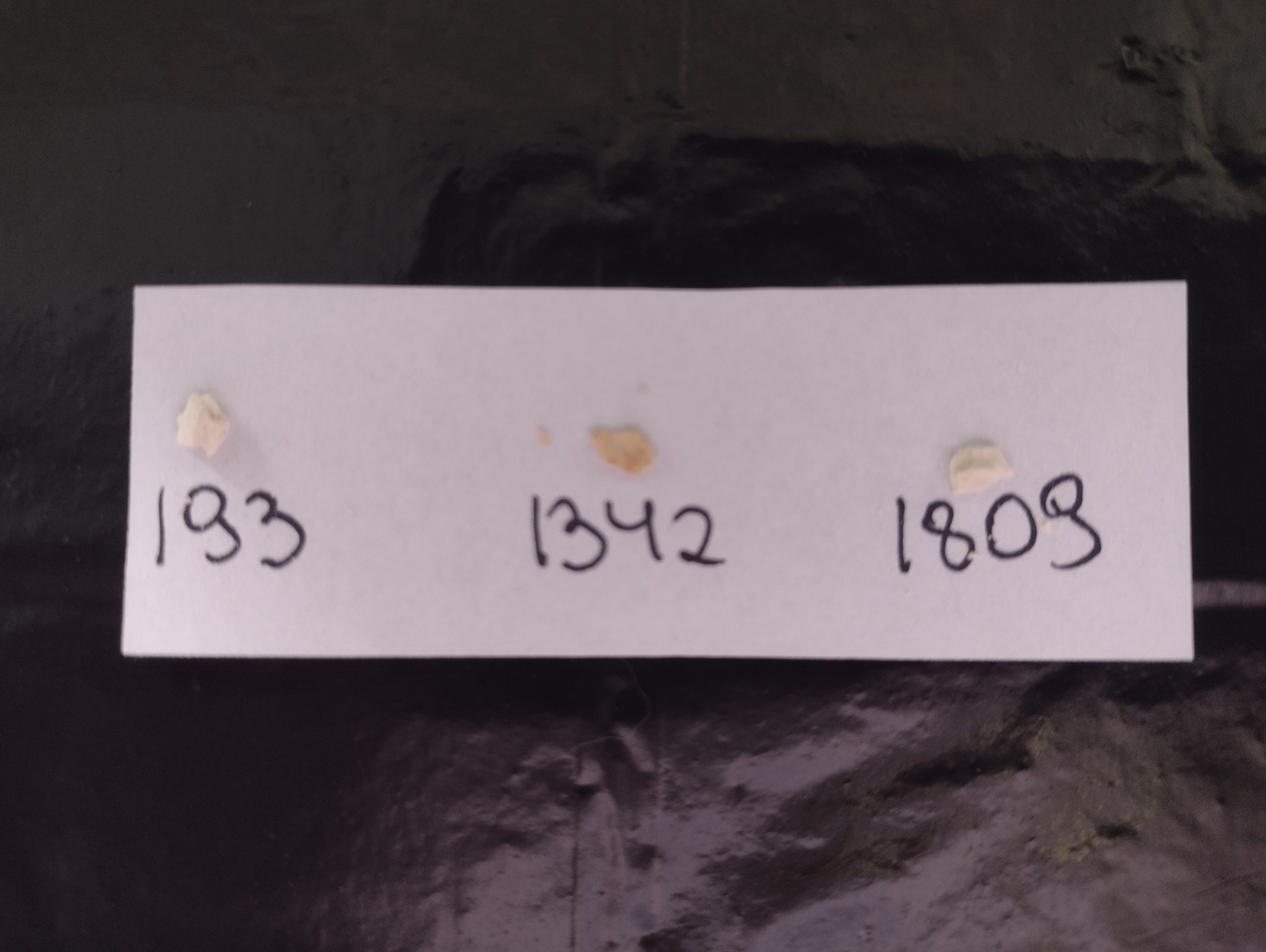


**Supplementary Figure S6.** Color of dried biosilica samples for TGA/DTA analysis: SZCZCH193 *N*. *wachnickianum*, SZCZM1342 *N. shiloi*, and SZCZP1809 *N.* cf. *shiloi*


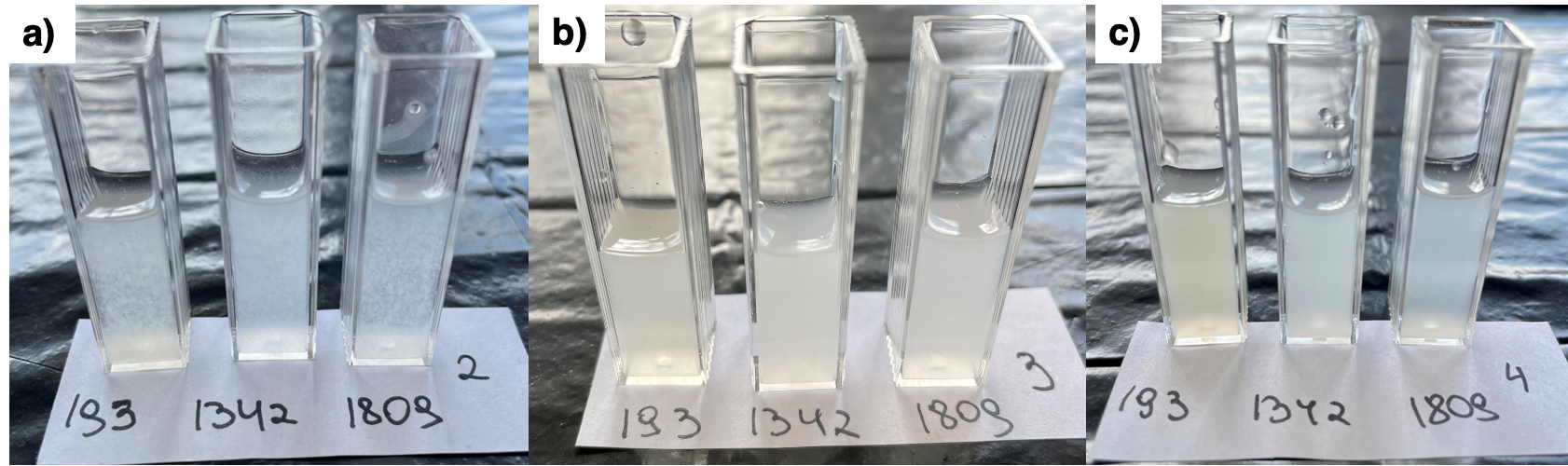


**Supplementary Figure S7.** Agglomeration of biosilica samples under (a) 2, (b) 3, (c) >4 pH in zeta potential measurements: SZCZCH193 *N*. *wachnickianum*, SZCZM1342 *N. shiloi*, and SZCZP1809 *N.* cf. *shiloi*

**Supplementary References**

1. Chandra, T.S. *et al.* Defatted algal biomass as a non-conventional low-cost adsorbent: surface characterization and methylene blue adsorption characteristics. *Bioresour. Technol*. **184**, 395-404 (2015).
2. Ahmed, M.N. & Ram, R.N. Removal of basic dye from waste-water using silica as adsorbent. *Environ. Pollut*. **77**, 79-86 (1992).
3. Han, R. *et al*. Study of equilibrium, kinetic and thermodynamic parameters about methylene blue adsorption onto natural zeolite. *Chem. Eng. J*. **145**, 496-504 (2009).
4. Touina, A. *et al*. Characterization and efficient dye discoloration of Algerian diatomite from Ouled Djilali-Mostaganem. *SN Appl. Sci*. **3**, 1-13 (2021).
5. Caparkaya, D. & Cavas, L. Biosorption of Methylene Blue by a Brown Alga Cystoseira barbatula Kützing. *Acta Chim. Slov*. **55**, 547-553 (2008).
6. Fu, Y. & Viraraghavan, T.Removal of a dye from an aqueous solution by the fungus Aspergillus niger. *Water Qual. Res. J*. **35**, 95-112 (2000).
7. Vilar, V.J., Botelho, C.M., & Boaventura, R.A. Methylene blue adsorption by algal biomass based materials: biosorbents characterization and process behaviour. *J. Hazard. Mater*. **147**, 120-132 (2007).
8. Van Eynde, E. *et al.* Effect of pretreatment and temperature on the properties of *Pinnularia* biosilica frustules. *RSC Adv*. **4**, 56200-56206; 10.1039/C4RA09305D (2014).
9. Lagergren, S. Zur theorie der sogenannten adsorption geloster stoffe. *Kungliga svenska vetenskapsakademiens. Handlingar* **24**, 1-39 (1898).
10. Blanchard, G., Maunaye, M., & Martin, G. Removal of heavy metals from waters by means of natural zeolites. *Water Res*. *18*, 1501-1507 (1984).
11. Aharoni, C. & Tompkins, F.C. Kinetics of adsorption and desorption and the Elovich equation, in *Advances in Catalysis, 21 (eds.* Eley, D.D., Pines, H., Weisz, P.B.) 1-49 (Academic Press Inc., 1970).
12. Weber Jr, W.J. & Morris, J.C. Kinetics of adsorption on carbon from solution. *J. Sanit. Eng. Div*. **89**, 31-59 (1963).
13. Boyd, G.E., Adamson, A.W. & Myers Jr, L.S. The exchange adsorption of ions from aqueous solutions by organic zeolites. II. Kinetics. *J. Am. Chem. Soc*. **69**, 2836-2848 (1947).
14. Bangham, A.D., Standish, M.M. & Watkins, J.C. Diffusion of univalent ions across the lamellae of swollen phospholipids. *J. Mol. Biol*. **13**, 238-252 (1965).
15. Langmuir, I. The adsorption of gases on plane surfaces of glass, mica and platinum. *J. Am. Chem. Soc*. **40**, 1361-1403 (1918).
16. Freundlich, H.M.F. Over the adsorption in solution. *J. Phys. Chem.* *57*, 385-471 (1906).
17. Sips, R. On the structure of a catalyst surface. *J. Phys. Chem*. *16*, 490-495 (1948).
